# Phylogenetic and Evolutionary Analysis of Porcine Epidemic Diarrhea Virus in Guangxi Province, China, during 2020 and 2024

**DOI:** 10.3390/v16071126

**Published:** 2024-07-14

**Authors:** Kaichuang Shi, Biao Li, Yuwen Shi, Shuping Feng, Yanwen Yin, Feng Long, Yi Pan, Yingyi Wei

**Affiliations:** 1 School of Basic Medical Sciences, Youjiang Medical University for Nationalities, Baise 533000, China; 2 College of Animal Science and Technology, Guangxi University, Nanning 530005, China; 3 Guangxi Center for Animal Disease Control and Prevention, Nanning 530001, China

**Keywords:** porcine epidemic diarrhea virus, S gene, M gene, N gene, phylogenetic analysis, recombination, evolution

## Abstract

The variant porcine epidemic diarrhea virus (PEDV) has caused considerable economic losses to the global pig industry since 2010. In this study, a total of 5859 diarrhea samples were collected from different pig farms in China’s Guangxi province during January 2020 and March 2024 and tested for PEDV using RT-qPCR. The positivity rate of PEDV was 11.90% (697/5859). Ninety-two PEDV-positive samples were selected based on sampling time, and the sampling region for amplification, sequencing, and analysis of the S1, M, and N genes. Phylogenetic analysis of the S1 gene revealed that all strains from Guangxi province were distributed in three subgroups, i.e., 81.5% (75/92) in the G2a subgroup, 4.3% (4/92) in the G2b subgroup, and 14.1% (13/92) in the G2c subgroup. The sequence analysis revealed that the S1 gene sequences from Guangxi province had higher homology with the variant strains than with the classical strains, showing as high as 99.2% with the variant strain AJ1102 and only 94.3% with the classical strain CV777. Recombination analysis revealed that the GX-BS08-2023 strain (G2c) from Guangxi province originated from inter-lineage recombination between the GX-BS09-2023 (G2a) and CH-JN547228-2011 (G1a) strains. In addition, the S1 gene of the G2a and G2b subgroup strains shared many mutations and insertions. There were common mutations of N143D and P235L in the G2a subgroup. Evolutionary analysis revealed that all Guangxi strains belonged to the G2 genotype. These strains have spread rapidly since the PEDV variant strains that emerged in 2010, weakened until 2021, and then remained stable. In conclusion, the results revealed the latest genetic evolution of circulating PEDV strains in Guangxi province in recent years, providing important information for preventing and controlling PEDV infection. Currently, the G2a subgroup strains are the predominant strains circulating in pig herds in Guangxi province, southern China.

## 1. Introduction

Porcine epidemic diarrhea virus (PEDV) is a novel enterovirus that causes severe gastrointestinal disease in infected pigs of all ages, especially less than 2-week-old piglets, and death in severe cases [1]. PEDV transmits through fecal–oral transmission, aerosol transmission, and vertical transmission, with a strong transmission capacity. Infected pigs show acute infectious gastroenteritis characterized by vomiting, watery diarrhea, dehydration, and anorexia. PEDV, as a member of the genus *Alphacoronavirus* in the *Coronaviridae* family, contains a genome (excluding polyA) measuring approximately 28 kb [2,3]. It was first discovered in the United Kingdom in 1971 [4]. In China, PEDV was first reported in the 1980s, and variant PEDV strains first appeared in 2010 [5]. The variant PEDV first appeared in the United States in 2013 and spread rapidly to Canada, Mexico, and Europe [6]. Since the genetic diversity of PEDV has constant variations, including deletions, insertions, and mutations, existing commercialized vaccines cannot induce sufficient mucosal immunity, resulting in their weak immune effect [7,8]. Moreover, due to the recombinant of live vaccine strains and wild-type strains, the clinical application of live vaccines might even produce variant PEDV strains with stronger virulence [9]. To date, PEDV has been reported worldwide [10,11]. At present, two genotypes of PEDV strains, G1 and G2 genotypes, are circulating in China [12].

The PEDV genome encodes ORF1a and ORF1b replicase polyproteins, spike (S), envelope (E), membrane (M), and nucleocapsid (N) structural proteins, and ORF3 accessory proteins [6,13]. S proteins can be divided into S1 (1–729 aa) and S2 (730–1387 aa) subunits. Of these proteins, S1, M, and N proteins play important roles. The S1 protein nanoparticles neutralize PEDV strains’ G1 and G2 genotypes [14]. A linear neutralizing epitope was found in S proteins, with potential use in the passive immunization of susceptible piglets against PEDV [15]. Furthermore, the PEDV S1 gene is continuously mutated, and the mutations are usually located in the N-terminal domain (NTD) (residues 21–324 aa based on the CV777 strain). Therefore, epidemiological investigations of the S1 gene are necessary [16,17]. The M protein is an important protein involved in viral infection, replication, and assembly, but its function remains to be elucidated [18]. N proteins also play important roles in viral replication and assembly, cell stress response to virus infection, the inhibition of type I interferon production, and signal transduction [19]. Therefore, S1, M, and N genes of PEDV were usually selected for molecular epidemiology to better understand the genetic diversity and evolution of the circulating PEDV strains.

So far, there have been some reports on PEDV epidemiological investigations, but most of them have selected complete genome and/or S gene (S1 gene) sequences for analysis [12,17,20,21,22]. In this study, a total of 5859 pig diarrhea samples were collected in Guangxi province from January 2020 to March 2024 for detecting PEDV using a quadruplex RT-qPCR [23], and 92 PEDV-positive samples were selected based on sampling time and region distribution for amplification, sequencing, and analysis of the S1, M, and N genes to monitor the genetic diversity and evolution of circulating PEDV strains in Guangxi province in recent years.

## 2. Materials and Methods

### 2.1. Clinical Samples

From January 2020 to March 2024, 5859 diarrheal fecal samples were collected from diarrheal piglets without vaccination in pig farms in Guangxi province, southern China. The samples were transported to the laboratory at ≤4 °C within 12 h post collection. The samples were resuspended using phosphate-buffered saline (PBS, pH 7.2) (1:4, *w*/*v*), vortexed (2 min), centrifuged (12,000 rpm, 5 min, 4 °C), and the total nucleic acids for detecting PEDV were extracted immediately or stored at −80 °C until use.

### 2.2. Amplification and Sequencing of Targeted Genes

The 5859 diarrheal fecal samples were tested using a quadruplex RT-qPCR developed in our laboratory [23]. A total of 697 (11.90%, 697/5859) samples were positive for PEDV. Then, according to the sampling location, sampling time, and Ct values (≤25 cycles), 92 PEDV-positive samples were selected for the amplification of S1 (2391 bp), M (682 bp), and N (1330 bp) genes using specific primers (Table 1). The total RNA was extracted, reverse transcribed to cDNA, and amplified in a 50 µL system according to the reported method [24] with minor modifications. The 50 µL system contained 2 × Taq PCR Master Mix 25 µL, forward/reverse primers (20 pmol/µL) of 0.8 µL each, cDNA of 5 µL, and nuclease-free distilled water of 18.4 µL. Different amplification procedures were used for different genes. S1 gene: 94 °C 3 min; 35 cycles of 94 °C 30 s, 52 °C 30 s, and 72 °C 90 s; 72 °C 10 min. M gene: 94 °C 3 min; 35 cycles of 94 °C 30 s, 50 °C 30 s, and 72 °C 50 s; 72 °C 10 min. N gene: 94 °C 3 min; 35 cycles of 94 °C 30 s, 58 °C 30 s, and 72 °C 90 s; 72 °C 10 min.

The PCR products were purified, cloned, and sequenced as per the procedures described in our previous report [24]. M and N gene sequences were obtained directly from sequencing. The complete S1 gene sequence was obtained by splicing and assembling the three gene fragment sequences. These sequences were further confirmed by NCBI BLAST analysis (https://blast.ncbi.nlm.nih.gov/Blast.cgi; accessed on 1 March 2024). Finally, 92 S1, 92 M, and 92 N gene sequences were obtained and sent to the NCBI GenBank database (Supplementary Tables S1–S3).

### 2.3. Sequence Comparison and Phylogenetic Analysis

The reference sequences, including 181 S1 sequences, 90 M sequences, and 90 N gene sequences, were downloaded from the NCBI GenBank website (https://www.ncbi.nlm.nih.gov/nucleotide/; accessed on 18 March 2024) (Supplementary Tables S1–S3). These sequences were derived from China, USA, Chesapeake, Mexico, Japan, Belgium, France, Germany, Thailand, Switzerland, Colombia, Ukraine, Canada, and other countries. In addition, 182 S1 gene sequences from Guangxi province were downloaded from the NCBI GenBank website (https://www.ncbi.nlm.nih.gov/nucleotide/; accessed on 18 March 2024) (Supplementary Table S4). The S1, M, and N gene sequences obtained in this study were compared to the above-downloaded gene sequences using the Clustal W algorithm of DNAstar 7.0 software (https://www.dnastar.com/software/; accessed on 18 March 2024). The genetic evolution tree was constructed using MEGA X 10.2.6 software (https://www.megasoftware.net/archived_version_active_download; accessed on 18 March 2024) and optimized through the Interactive Tree Of Life (iTOL) (https://itol.embl.de/; accessed on 24 March 2024) [25]. In addition, a genetic evolutionary tree was constructed using 92 obtained S1 gene sequences and 182 reference S1 gene sequences from Guangxi province (Supplementary Table S4).

### 2.4. Bayesian Temporal Dynamics Analysis

MEGA X 10.2.6 software was used to rematch reference sequences and sequences obtained from Guangxi in this study. Then, the model was selected using the maximum likelihood method of IQ-TREE1.6.12 software (http://iqtree.cibiv.univie.ac.at/; accessed on 24 March 2024). The bootstrap value was set to 1000, and the genetic evolution tree was reconstructed. To determine the time structure, TempEst 1.5.3 was used for a regression verification of the root tip genetic distance of the sequences [26,27]. Bayesian Markov Chain Monte Carlo (MCMC) was selected to infer the dispersion time of the PEDV S1 gene in BEAST v1.10.4 (http://beast.community/; accessed on 24 March 2024). Then, we calculated the appropriate alternative model using ModelFinder in PhyloSuite software [28], selected the molecular clock replacement model and Bayesian skyline replacement model, ran 200 million steps in parallel on three chains, aged by 10%, and visualized the data with ESS > 200 of all parameters after operation through Tracer software. All data were annotated by TreeAnnotator 1.10.4 software (http://beast.community/programs; accessed on 24 March 2024) after aging by 10% and visualized using Fig Tree version 1.4.4 software (http://beast.community/figtree; accessed on 24 March 2024).

### 2.5. Recombination Events Analysis

A recombinant analysis was performed as per the previous report [24]. All S1 gene sequences were analyzed using the Recombination Detection Program (RDP4) software (http://www.bioinf.manchester.ac.uk/recombination/programs; accessed on 28 March 2024), and the SimPlot 3.5.1 software (https://github.com/Stephane-S/Simplot_PlusPlus; accessed on 8 March 2024) was used to verify the potentially recombinant sequence.

### 2.6. Amino Acid Difference Analysis of PEDV S1 Gene

To analyze genetic identity between different subgroups, Bioedit v.7.2.5 software (https://bioedit.software.informer.com/download/; accessed on 15 March 2024) was used to analyze S1 gene sequences. The deduced S1 amino acid sequences of representative strains from each subgroup were selected and compared to sequences obtained in this study according to genetic evolution tree results. The CV777 strain (Accession No. AF353511) [29] and AJ1102 strain (Accession No. JX188454) [30] were used as typical representative strains of classical and variant PEDV, respectively. The amino acid sites of the main reference strains were analyzed and compared.

## 3. Results

### 3.1. Test Results of Clinical Samples

**Figure 1:**
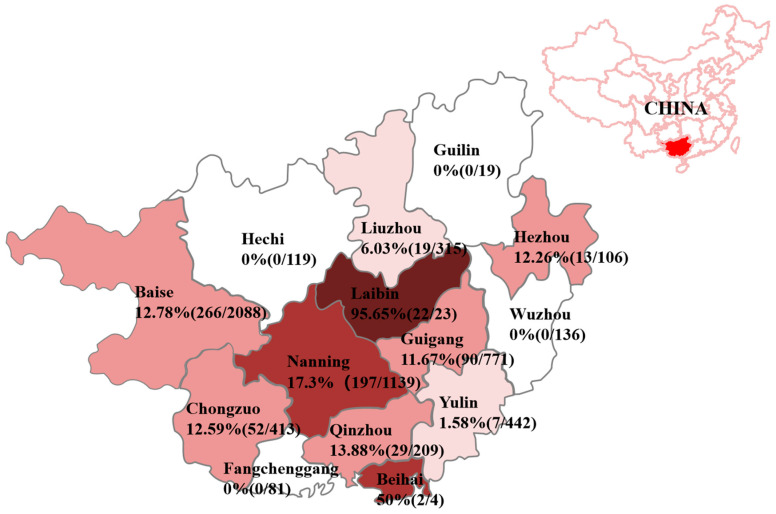
PEDV distribution in different regions of Guangxi province. Color depths are marked according to the positivity rate of each region.

The 5859 clinical samples were detected for PEDV by a quadruplex RT-qPCR [23], and the positivity rate of PEDV was 11.90% (697/5859). The positivity rates were 9.76% (36/369), 4.62% (41/887), 13.88% (386/2780), 15.54% (211/1358), and 4.95% (23/465) from 2020 to 2024, respectively. Figure 1 shows the distribution of 697 positive samples from Guangxi province. Ninety-two positive samples were selected based on sampling time, sampling locations, and detected Ct values for S1, M, and N gene sequence analysis. Finally, 92 S1, 92 M, and 92 N gene sequences were obtained. These were uploaded to the NCBI GenBank under the following accession numbers: OR659219-OR659270 and PP460558-PP460597 for the S1 gene, OR659271-OR659322 and PP460598-PP460637 for the M gene, and OR659323-OR659374 and PP460638-PP460677 for the N gene. The homology of S1, M, and N gene sequences with the classical strain CV777 and variant strain AJ1102 is shown in Table 2. The homology of these gene sequences between Guangxi’s strains and other countries’ strains is shown in Table 3.

### 3.2. Phylogenetic Analysis Based on S1 Gene Sequences

**Figure 2:**
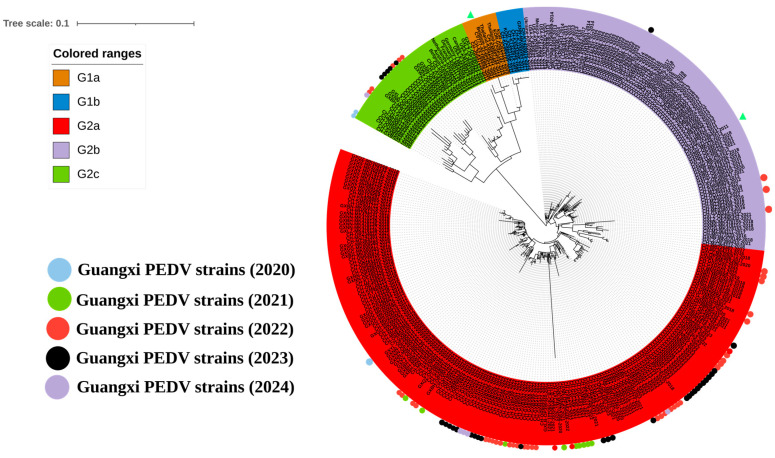
A phylogenetic tree based on PEDV S1 gene nucleotide sequences. Different colored circles label the sequences according to the year they were obtained. The classical strain CV777 and variant strain AJ1102 are labeled with triangles.

To analyze genetic characteristics, 92 S1 gene sequences from this study and 181 S1 gene sequences downloaded from the NCBI GenBank were used to construct a genetic evolution tree (Figure 2). The results showed that the sequences from Guangxi province were distributed in the G2 genotype, of which 81.5% (75/92), 4.3% (4/92), and 14.1% (13/92) belonged to the G2a, G2b, and G2c subgroups, respectively. Based on these findings, PEDV strains from the G2a subgroup were the predominant strains circulating in Guangxi province.

### 3.3. Phylogenetic Analysis Based on S1 Gene Sequences from Guangxi Province

**Figure 3:**
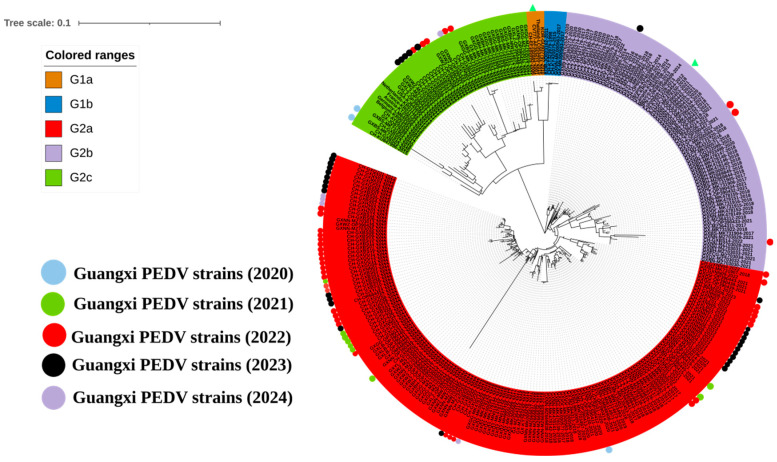
A phylogenetic tree based on PEDV S1 gene nucleotide sequences from Guangxi province. Colored circles were used to label the sequences according to the year they were obtained. The classical strain CV777 and variant strain AJ1102 are labeled with triangles.

To further analyze the genetic evolution of the PEDV S1 gene in Guangxi province, 182 S1 gene sequences from Guangxi province were downloaded from the NCBI GenBank. The earliest sequence can be traced back to 2011 (Accession No. JQ979288). Based on the genetic evolutionary tree of the S1 gene (Figure 3), representative sequences were selected from different subgroups to reconstruct the genetic evolution tree of the PEDV S1 gene in Guangxi province. The results revealed that the 182 PEDV S1 gene sequences from Guangxi province from 2011 to 2024 and the 92 S1 gene sequences obtained in this study were mainly distributed in the G2 genotype, with 161 strains in the G2a subgroup, 71 strains in the G2b subgroup, 40 strains in the G2c subgroup, and only 2 strains distributed in the G1b subgroup (Figure 3, Table 4).

### 3.4. Phylogenetic Analysis Based on M Gene Sequences

**Figure 4:**
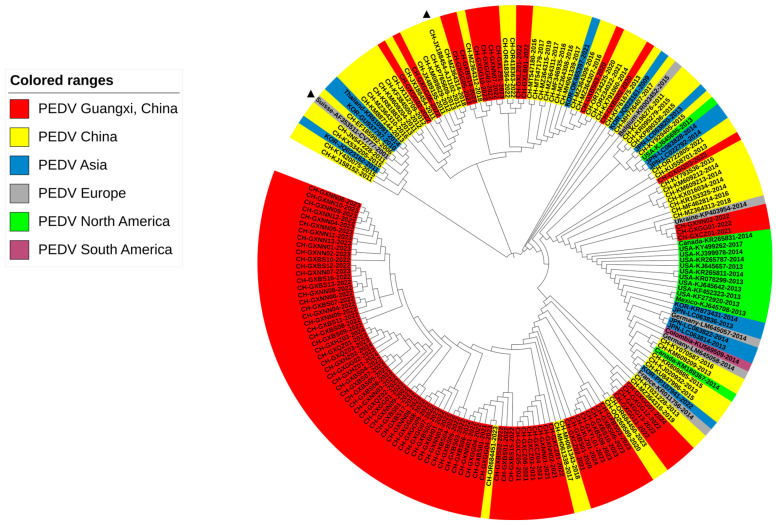
A phylogenetic tree based on PEDV M gene nucleotide sequences. The classical strain CV777 and variant strain AJ1102 are labeled with triangles.

The 92 M gene sequences obtained in this study and 90 M gene sequences downloaded from the NCBI GenBank were used to construct the genetic evolution tree. Our results showed that Chinese sequences had a wide distribution range, and most of Guangxi’s sequences were located in the same subgroup of sequences from other Chinese provinces. However, a few sequences were located in the subgroup of sequences from other countries (Figure 4). No sequence from Guangxi province was found in the same subgroup as classical strain CV777.

### 3.5. Phylogenetic Analysis Based on N Gene Sequences

**Figure 5:**
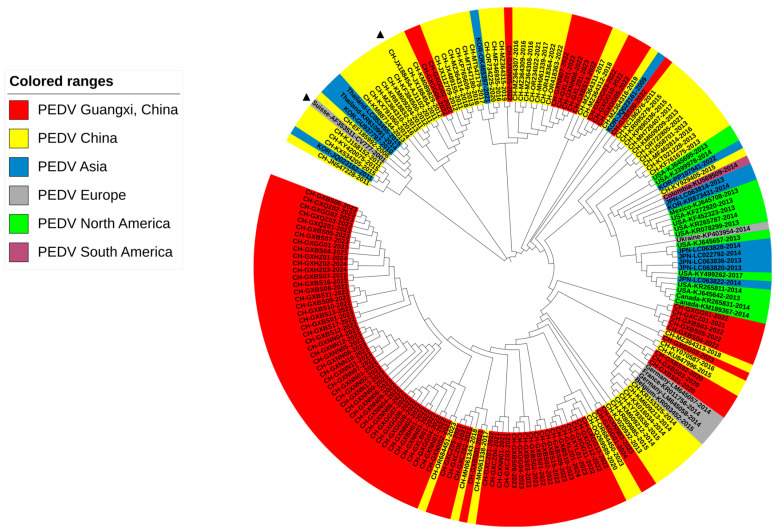
A phylogenetic tree based on PEDV N gene nucleotide sequences. The classical strain CV777 and variant strain AJ1102 are labeled with triangles.

The 92 N gene sequences obtained in this study and 90 N gene sequences downloaded from the NCBI GenBank were used to construct the genetic evolution tree (Figure 5). Our results showed that most sequences obtained in Guangxi were distributed in the same subgroups as other provinces in China, France, Germany, and Belgium. This result was significantly different from genetic evolution trees constructed based on S1 and M gene sequences. However, other N gene sequences obtained in this study were also distributed in the subgroups of the variant strain. However, they were not in the same subgroup as the classical strain CV777.

### 3.6. Bayesian Temporal Dynamics Analysis

**Figure 6:**
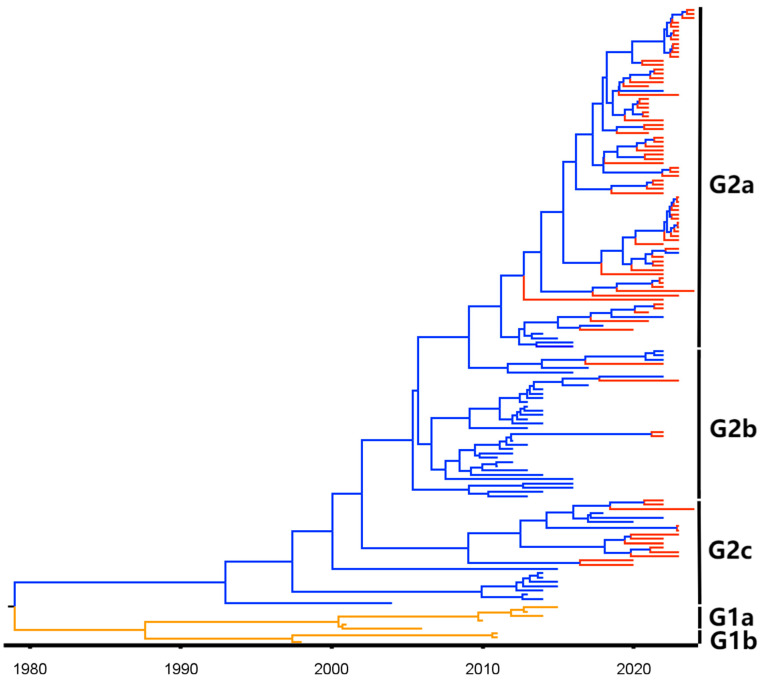
The maximum clade credibility (MCC) tree based on PEDV S1 gene nucleotide sequences. The G1 and G2 genotypes are labeled orange and blue, respectively. The obtained sequences from Guangxi province are labeled in red.

**Figure 7:**
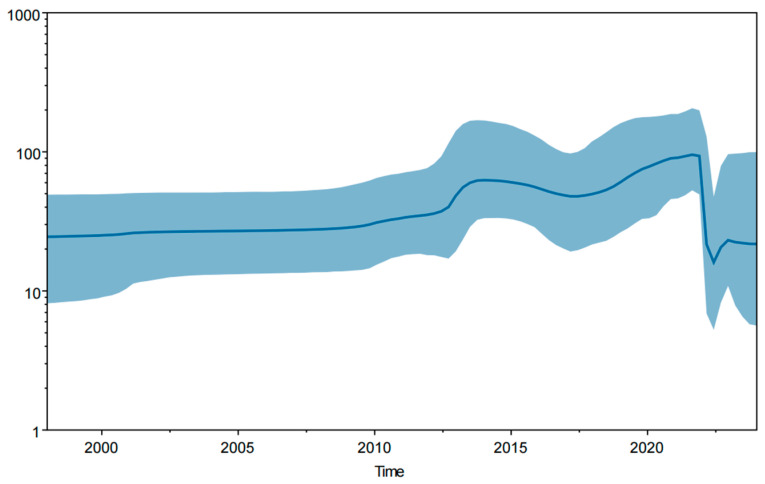
A Bayesian skyline of PEDV S1 gene sequences in Guangxi province. The genetic diversity average is indicated by a dark blue line, and the 95% confidence interval is indicated by light blue shading.

The MCC tree was constructed based on the PEDV S1 gene and the PEDV temporal scale (Figure 6). It indicated that all PEDV strains could be divided into five subgroups: G1a, G1b, G2a, G2b, and G2c (Figure 6). The sequence differentiation times for Guangxi province were after 2010. All the strains were located in the G2 genotype, which belongs to variant strains. The Bayesian skyline (Figure 7) provides a practical population size map of PEDV transmission in Guangxi province in recent years. The virus has spread rapidly since PEDV variant strains emerged in 2010, weakening until 2021 before maintaining a stable trend.

### 3.7. Recombination Analysis of the PEDV S1 Gene

**Figure 8:**
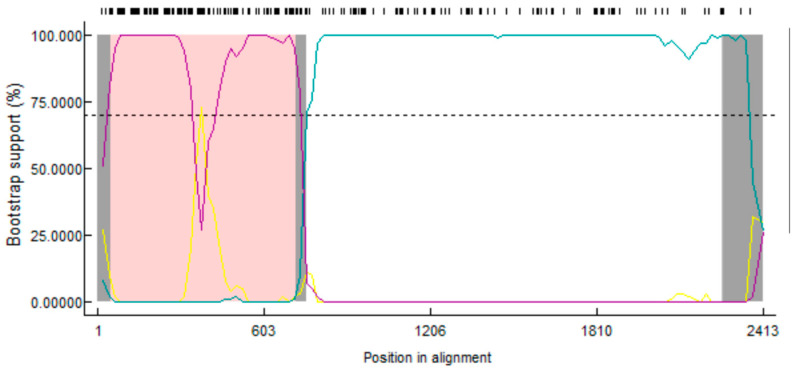
Recombination analysis using RDP4 software. Recombination event analysis of the S1 gene of the GX-BS08-2023 strain (G2c) for potential recombination events between the GX-BS09-2023 (G2a) and CH-JN547228-2011 (G1a) strains.

Recombination analysis based on the S1 gene indicated that one strain from Guangxi province showed recombination signals (Figure 8). The GX-BS08-2023 strain (G2c) originated from a recombination between the GX-BS09-2023 (G2a) and CH-JN547228-2011 (G1a) strains. Among them, the primary parent (GX-BS09-2023 strain) showed 99.4% similarity, the minor parent (CH-JN547228-2011 strain) showed 92.9% similarity, and potential breakpoints were located in the 50 nt–718 nt region.

### 3.8. Genetic Evolution of PEDV S1, M, and N Genes

The genetic evolution rates of PEDV S1, M, and N genes from Guangxi were analyzed using Tracer v1.7.1 software (https://github.com/beast-dev/tracer/releases/latest; accessed on 20 March 2024) (Table 5) and showed 1.527 × 10^−3^, 1.518 × 10^−4^, and 1.076 × 10^−3^ substitutions/site/year (S/S/Y) for the S1, M, and N genes, respectively.

### 3.9. Amino Acid Sequence Analysis of the S1 Protein

This study’s S1 gene amino acid sequences from Guangxi province and other amino acid sequences from each subgroup were imported into BioEdit software Version 7.2.5 (http://www.ebi.ac.uk/Tools/clustalw2/; accessed on 18 March 2024) to analyze the genetic identity between different subgroups. The results showed that amino acid variations, including mutation, deletion, and insertion, were found in PEDV strains in the G2 genotype. Compared to the classical strain CV777, common mutations were found in the G2 genotype from Guangxi province, including D319Q, E374Q, S466A, T561S, L624F, I647V, N736S, and Y778S (Supplementary Figure S1). Furthermore, the G2a and G2b subgroup strains showed common mutations of S28A, M56G, S64T, I71H, D86R, I124T, D134S, N143D, K166S, A183S, L191F, R201K, R206G, T215E, and E234Q; insertions of 58NQGV61 and 144N; and a deletion of 167–168 aa. However, there were different mutation sites in the G2a (N143D) and G2b (L82V) subgroups. In addition, the G2c subgroup showed mutations in I71L, S122G, R163Q, D167N, and R205K (Supplementary Figure S1).

## 4. Discussion

Since 2010, PEDV has caused significant economic losses to the pig industry worldwide. It is challenging to prevent and control due to the strong virulence and high mutation rate of circulating G2 genotype strains [31]. At present, PEDV has strong transmission characteristics. Furthermore, the classical strain CV777 vaccine used in clinical practice is not fully effective against variant PEDV strains circulating in China. Therefore, PEDV is still an important threat to the Chinese pig industry [32,33]. Of the 5859 diarrhea samples collected in Guangxi province from January 2020 to March 2024, 697 (11.90%) samples tested positive for PEDV, which differed from the 53.94% positivity rate reported in clinical samples collected in Guangxi province between 2017 and 2022 [20]. This difference might be attributed to the wide use of attenuated and/or inactivated PEDV vaccines and the implementation of strict biosecurity measures. In recent years, PEDV vaccines have been continuously improved to achieve better immune effects. They have been widely used in pig farms to increase pig herds’ overall immunity [10,34,35]. In addition, the outbreak of African swine fever (ASF) in 2018 in China led to more comprehensive biosafety systems and stricter biosecurity measures on pig farms, which greatly reduced the spread of pathogens between pig herds [36,37,38]. In this study, the positivity rates of clinical samples were 9.76%, 4.62%, 13.88%, 15.54%, and 4.95% from 2020 to 2024, respectively. These results indicate that the PEDV positivity rate in Guangxi province has decreased significantly in recent years. However, PEDV still maintains a certain infection rate requiring continuous prevention and control.

Guangxi province is located in southern China and borders various Southeast Asian countries. It is an important port for pig and pig production trade between China and Vietnam. Consequently, swine pathogens may spread between the two countries, increasing the diversity and complexity of PEDV epidemic strains in Guangxi province. Currently, PEDV has two genotypes: G1 (G1a, G1b) and G2 (G2a, G2b, and G2c) [21,39,40]. In this study, 92 S1 gene sequences obtained in Guangxi province belonged to the G2 genotype. The G2 genotype has stronger virulence than the G1 genotype [41], and current commercial vaccines cannot provide complete protection against it [7,8]. It has been reported that the full-length PEDV S gene mRNA vaccine confers protection against PEDV infection in immunized piglets, suggesting an important path forward for PEDV prevention and control [42].

Sequence analysis revealed that gene sequences from Guangxi province had higher homology with variant strain AJ1102 than classical strain CV777 (Table 2). Of the S1 gene sequences from Guangxi province analyzed in this study, there were 2 G1b strains, 161 G2a strains, 71 G2b strains, and 40 G2c strains, indicating that G2a strains are the main subgroup currently circulating in Guangxi province (Table 4). This finding further confirms that G2 genotype strains have become more prevalent in China in recent years [10,43]. Moreover, the M and N gene sequence analysis revealed that all sequences from Guangxi were located in the same clade as the AJ1102 variant strain. This finding confirmed that the current circulating strains in Guangxi province are closely related to the variant strains [20,44].

According to the results of the MCC tree constructed after Bayesian analysis, the sequence differentiation time of a strain (GXNN04-2022; GenBank accession No. OR659265) from Guangxi province in the G2a subgroup was earlier than other strains from Guangxi province. It is speculated that G2a subgroup strains have existed for a long time in Guangxi province, which may account for why G2a subgroup strains are the dominant strains circulating in Guangxi province. Other scientists have also analyzed the molecular characteristics and pathogenicity of G2a subgroup strains from Guangxi province in recent years [20,44,45]. The results were important to our understanding of PEDV in Guangxi province. The Bayesian skyline showed the effective population scale transmission of PEDV; our results indicated that PEDV transmission had a significant downward trend in the past two years before leveling off. Since 2000, virus species diversity has maintained a slow rise, then an upward trend after a slow rise in 2012. Until around 2021, the virus showed a significant decline and then spread steadily, which differs from Bai et al.’s results [20]. The most likely reason is to reduce the use of vaccines. Since the outbreak of ASF in 2018, most pig farms in China have implemented more comprehensive biosafety systems and stricter biosecurity measures, and the positivity rate of PEDV in clinical samples has gradually decreased in recent years. Therefore, they reduced or stopped using PEDV vaccines. This situation reduced the immune pressure of clinical epidemic strains and slowed down the rate of genetic variation in PEDV.

One of the most important ways coronaviruses evolve is through recombination [46,47]. Recombinant analysis revealed that the GX-BS08-2023 (G2c) strain indicated a recombination between the GX-BS09-2023 (G2a) and CH-JN547228-2011 (G1a) strains. Interestingly, the recombinant strain GX-BS08-2023 had primary and secondary parents from different subgroups, but a higher homology with GX-BS09-2023, which belonged to the G2a subgroup. Other studies suggested that the G2c subgroup may have evolved from recombination [39]. The results of this study also indicated that recombination was an important evolutionary model of PEDV and may be one of the reasons for the genetic diversity of circulating strains in Guangxi province.

In addition, mutation, deletion, and insertion of amino acids in the PEDV S1 gene were also found in Guangxi province (Supplementary Figure S1). Strains with a large deletion in the S gene and/or ORF1a gene have been discovered worldwide [9,48,49]. Variation in the S1 gene is considered the main cause of changes in the virulence of the virus [50,51]. PEDV S1 protein has a high mutation rate, and some studies have found new mutations and insertions in the S1 protein [16,17,52]. In this study, mutation, deletion, and insertion in many PEDV S1 gene locations were also found, which might affect viral virulence and account for why PEDV is difficult to prevent and control in Guangxi province.

## 5. Conclusions

The clinical diarrheal samples from Guangxi province in southern China from January 2020 and March 2024 showed an 11.90% positivity rate for PEDV. Sequence analysis of S1, M, and N genes revealed that all 92 obtained strains belonged to the G2 genotype and had high homology with variant strain AJ1102. Bayesian skyline results showed that the effective population size of the virus decreased significantly in 2021, but then maintained a stable trend. Recombination analysis showed that the G2c subgroup might have originated from recombination. Sequence analysis revealed that the S1 gene of G2a and G2b subgroup strains shared many mutations and insertions. In addition, the G2a subgroup had common mutations of N143D and P235L. The data provided the latest epidemic situation for PEDV in Guangxi province and important information on the genetic diversity and evolution of the circulating strains in pig herds.

## Figures and Tables

**Table 1:** The amplification primers for PEDV S1, M, and N genes.

Gene	Primer	Sequence (5′→3′)	Product/bp
S1	PEDV-SI-F PEDV-SI-R PEDV-SII-F PEDV-SII-R PEDV-SIII-F PEDV-SIII-R	CCATTAGTGATGTTGTGTTAG GCACAGCAGCTCCATT CCACATACCAGAAGGTTTTAG CCAGTAATCAACTCACCCTT CCTGAGTTTGGTAGTGGTGTTA GGTGAGTAATTGTTTACAACGAGAG	1040 1146 654
M	PEDV-M-F PEDV-M-R	GTCTTACATGCGAATTGACC GGCATAGAGAGATAATGGCA	808
N	PEDV-N-F PEDV-N-R	TGCGGTTCTCACAGATAGTG AAGTCGCTAGAAAAACACTCAGTAAT	1462

**Table 2:** Homology of S1, M, and N gene sequences from Guangxi province with classical strain CV777 and variant strain AJ1102.

Guangxi Strain (n = 92)		Percentage of Nucleotide (Amino Acid) Identity (%)	
		CV777	AJ1102
S1	G2a (n = 75)	91.1–92.0% (89.4–90.9%)	96.9–98.8% (96.5–98.1%)
	G2b (n = 4)	91.2–92.0% (89.1–90.9%)	96.3–99.2% (95.6–99.1%)
	G2c (n = 13)	93.6–94.3% (92.4–94.0%)	92.0–92.6% (90.5–92.1%)
M		96.6–98.1% (93.6–99.4%)	97.1–99.6% (93.6–99.4%)
N		94.8–95.8% (80.2–93.8%)	95.3–99.7% (82.7–95.6%)

**Table 3:** Homology of S1, M, and N gene sequences from Guangxi province and other strains from different countries.

Guangxi Strain (n = 92)	Percentage of Nucleotide (Amino Acid) Identity (%)		
	The Strains Obtained in This Study	The Other Strains from Guangxi Province	The Other Strains from China and Other Countries
S1	91.8–99.9% (89.3–99.5%)	91.2–99.9% (89.0–99.5%)	90.8–99.8% (88.3–99.4%)
M	97.0–100.0% (96.2–100.0%)	96.6–99.9% (92.3–100.0%)	95.1–99.9% (84.3–100.0%)
N	95.3–99.9% (82.6–100.0%)	95.1–99.9% (77.3–100.0%)	93.8–99.7% (73.3–100.0%)

**Table 4:** The distribution of PEDV strains from Guangxi province in different subgroups based on the S1 gene genetic evolution tree.

Year	G1b	G2a	G2b	G2c	Total
2011	0	0	0	1	1
2015	0	1	0	0	1
2016	0	0	4	0	4
2017	2	11	8	1	22
2018	0	27	26	11	64
2019	0	5	1	3	9
2020	0	13	10	2	25
2021	0	39	17	8	64
2022	0	35	4	8	47
2023	0	26	1	5	32
2024	0	4	0	1	5
**Total**	2	161	71	40	274

**Table 5:** Evolutionary rates of PEDV S1, M, and N genes.

Gene	Evolutionary Rate (S/S/Y)	95% HPD Interval
S1	1.527 × 10^−3^	1.2014 × 10^−3^–1.8418 × 10^−3^
M	1.518 × 10^−4^	1.269 × 10^−4^–1.7983 × 10^−4^
N	1.076 × 10^−3^	8.2589 × 10^−4^–1.3442 × 10^−3^

## Data Availability

Data are contained within the article and Supplementary Materials.

## Supplementary Materials

The following supporting information can be downloaded at: https://www.mdpi.com/article/10.3390/v16071126/s1, Table S1: Information on the S1 gene of PEDV strains used in this study. Table S2: Information on the M gene of PEDV strains used in this study. Table S3: Information on the N gene of PEDV strains used in this study. Table S4: Information on the S1 gene of PEDV strains from Guangxi province used in this study. Figure S1: Amino acid difference diagrams for the PEDV S1 protein of different subgroups.
