# Supplementary material for: Phylogenetic and Evolutionary Analysis of Porcine Epidemic Diarrhea Virus in Guangxi Province, China, during 2020 and 2024
Source: Viruses. 2024 Jul 14;16(7):1126. doi: 10.3390/v16071126 (PMC11281377; doi:10.3390/v16071126)
Supplement: Supplementary file 1 [file viruses-16-01126-s001.zip › Supplementary Table S1-S4.pdf]

Supplementary

Supplementary Table S1. The information on S1 gene of PEDV strains used in this study.

Virus Strain	Date	Origin	Accession No.	Virus Strain	Date	Origin	Accession No.
CH-GXBS01-2020	2020	Baise, GX, CHN	OR659220	CV777-AF353511-2001	2001	Suisse	AF353511
CH-GXBS02-2020	2020	Baise, GX, CHN	OR659221	AJ1102-JX188454-2012	2012	CHN	JX188454
CH-GXBS03-2020	2020	Baise, GX, CHN	OR659222	GXCZ-MK000564-2017	2017	Chongzuo, GX, CHN	MK000564
CH-GXBS01-2021	2021	Baise, GX, CHN	OR659223	GXGG-MK000576-2017	2017	Guigang, GX, CHN	MK000576
CH-GXCZ01-2021	2021	Chongzuo, GX, CHN	OR659240	GXNN-MK000563-2017	2017	Nanning, GX, CHN	MK000563
CH-GXCZ02-2021	2021	Chongzuo, GX, CHN	OR659241	GXNN-MK000578-2017	2017	Nanning, GX, CHN	MK000578
CH-GXCZ03-2021	2021	Chongzuo, GX, CHN	OR659242	GXQZ-MH985745-2017	2017	Qinzhou, GX, CHN	MH985745
CH-GXCZ04-2021	2021	Chongzuo, GX, CHN	OR659243	GXQZ-MK000569-2017	2017	Qinzhou, GX, CHN	MK000569
CH-GXCZ05-2021	2021	Chongzuo, GX, CHN	OR659244	GXBH-MK000572-2018	2018	Beihai, GX, CHN	MK000572
CH-GXCZ06-2021	2021	Chongzuo, GX, CHN	OR659245	GXBH-MK000573-2018	2018	Beihai, GX, CHN	MK000573
CH-GXNN01-2021	2021	Nanning, GX, CHN	OR659260	GXBH-MK478139-2018	2018	Beihai, GX, CHN	MK478139
CH-GXNN02-2021	2021	Nanning, GX, CHN	OR659261	GXBH-MK478148-2018	2018	Beihai, GX, CHN	MK478148
CH-GXBH01-2022	2022	Beihai, GX, CHN	OR659219	GXCZ-MK000571-2018	2018	Chongzuo, GX, CHN	MK000571
CH-GXBS01-2022	2022	Baise, GX, CHN	OR659224	GXGG-MK000579-2018	2018	Guigang, GX, CHN	MK000579
CH-GXBS02-2022	2022	Baise, GX, CHN	OR659225	GXGG-MK000583-2018	2018	Guigang, GX, CHN	MK000583
CH-GXBS03-2022	2022	Baise, GX, CHN	OR659226	GXGG-MK478145-2018	2018	Guigang, GX, CHN	MK478145
CH-GXBS04-2022	2022	Baise, GX, CHN	OR659227	GXGG-MK478153-2018	2018	Guigang, GX, CHN	MK478153
CH-GXBS05-2022	2022	Baise, GX, CHN	OR659228	GXGG-MK478154-2018	2018	Guigang, GX, CHN	MK478154
CH-GXBS06-2022	2022	Baise, GX, CHN	OR659229	GXLB-MK478152-2018	2018	Laibin, GX, CHN	MK478152
CH-GXBS07-2022	2022	Baise, GX, CHN	OR659230	GXLZ-MK478138-2018	2018	Liuzhou, GX, CHN	MK478138

CH-GXBS08-2022	2022	Baise, GX, CHN	OR659231	GXNN-MK478142-2018	2018	Nanning, GX, CHN	MK478142
CH-GXBS09-2022	2022	Baise, GX, CHN	OR659232	GXNN-MK478149-2018	2018	Nanning, GX, CHN	MK478149
CH-GXBS10-2022	2022	Baise, GX, CHN	OR659233	GXNN-MK478155-2018	2018	Nanning, GX, CHN	MK478155
CH-GXBS11-2022	2022	Baise, GX, CHN	OR659234	GXQZ-MN019125-2018	2018	Qinzhou, GX, CHN	MN019125
CH-GXBS12-2022	2022	Baise, GX, CHN	OR659235	GXQZ-MK478143-2018	2018	Qinzhou, GX, CHN	MK478143
CH-GXBS13-2022	2022	Baise, GX, CHN	OR659236	GXQZ-MK478144-2018	2018	Qinzhou, GX, CHN	MK478144
CH-GXBS14-2022	2022	Baise, GX, CHN	OR659237	GXYL-MK000581-2018	2018	Yulin, GX, CHN	MK000581
CH-GXBS15-2022	2022	Baise, GX, CHN	OR659238	GXYL-MK000584-2018	2018	Yulin, GX, CHN	MK000584
CH-GXBS16-2022	2022	Baise, GX, CHN	OR659239	GXBH-MW114972-2019	2019	Beihai, GX, CHN	MW114972
CH-GXBS17-2022	2022	Baise, GX, CHN	PP460558	GXBH-MW703493-2019	2019	Beihai, GX, CHN	MW703493
CH-GXCZ01-2022	2022	Chongzuo, GX, CHN	OR659246	GXQZ-MN019126-2019	2019	Qinzhou, GX, CHN	MN019126
CH-GXGG01-2022	2022	Guigang, GX, CHN	OR659247	GXNN-OP390412-2020	2020	Nanning, GX, CHN	OP390412
CH-GXGG02-2022	2022	Guigang, GX, CHN	OR659248	GXNN-MW703495-2020	2020	Nanning, GX, CHN	MW703495
CH-GXGG03-2022	2022	Guigang, GX, CHN	OR659249	GXNN-MW703496-2020	2020	Nanning, GX, CHN	MW703496
CH-GXGG04-2022	2022	Guigang, GX, CHN	OR659250	GXNN-MW703497-2020	2020	Nanning, GX, CHN	MW703497
CH-GXGG05-2022	2022	Guigang, GX, CHN	OR659251	GXNN-MW703498-2020	2020	Nanning, GX, CHN	MW703498
CH-GXGG06-2022	2022	Guigang, GX, CHN	OR659252	GXQZ-OP390413-2020	2020	Qinzhou, GX, CHN	OP390413
CH-GXGG07-2022	2022	Guigang, GX, CHN	OR659253	GXWZ-MW703494-2020	2020	Wuzhou, GX, CHN	MW703494
CH-GXGG08-2022	2022	Guigang, GX, CHN	OR659254	GXYL-OP390414-2020	2020	Yulin, GX, CHN	OP390414
CH-GXGG09-2022	2022	Guigang, GX, CHN	OR659255	GXBH-OP390417-2021	2021	Beihai, GX, CHN	OP390417
CH-GXGG10-2022	2022	Guigang, GX, CHN	OR659256	GXGG-OP390431-2021	2021	Guigang, GX, CHN	OP390431
CH-GXGG11-2022	2022	Guigang, GX, CHN	OR659257	GXGG-OP390443-2021	2021	Guigang, GX, CHN	OP390443
CH-GXGG12-2022	2022	Guigang, GX, CHN	OR659258	GXLZ-OP390424-2021	2021	Liuzhou, GX, CHN	OP390424
CH-GXGG13-2022	2022	Guigang, GX, CHN	OR659259	GXLZ-OP390425-2021	2021	Liuzhou, GX, CHN	OP390425
CH-GXLB01-2022	2022	Laibin, GX, CHN	PP460559	GXLZ-OP390430-2021	2021	Liuzhou, GX, CHN	OP390430
CH-GXLZ01-2022	2022	Liuzhou, GX, CHN	PP460560	GXLZ-OP390433-2021	2021	Liuzhou, GX, CHN	OP390433

CH-GXNN01-2022	2022	Nanning, GX, CHN	OR659262	GXLZ-OP390434-2021	2021	Liuzhou, GX, CHN	OP390434
CH-GXNN02-2022	2022	Nanning, GX, CHN	OR659263	GXLZ-OP390448-2021	2021	Liuzhou, GX, CHN	OP390448
CH-GXNN03-2022	2022	Nanning, GX, CHN	OR659264	GXNN-OP390426-2021	2021	Nanning, GX, CHN	OP390426
CH-GXNN04-2022	2022	Nanning, GX, CHN	OR659265	GXNN-OP390428-2021	2021	Nanning, GX, CHN	OP390428
CH-GXNN05-2022	2022	Nanning, GX, CHN	OR659266	GXNN-OP390453-2021	2021	Nanning, GX, CHN	OP390453
CH-GXNN06-2022	2022	Nanning, GX, CHN	OR659267	GXQZ-OP390445-2021	2021	Qinzhou, GX, CHN	OP390445
CH-GXNN07-2022	2022	Nanning, GX, CHN	OR659268	GXWZ-OP390422-2021	2021	Wuzhou, GX, CHN	OP390422
CH-GXNN08-2022	2022	Nanning, GX, CHN	OR659269	GXWZ-OP390429-2021	2021	Wuzhou, GX, CHN	OP390429
CH-GXNN09-2022	2022	Nanning, GX, CHN	OR659270	GXNN-OP390449-2022	2022	Nanning, GX, CHN	OP390449
CH-GXBS01-2023	2023	Baise, GX, CHN	PP460561	GXNN-OP390450-2022	2022	Nanning, GX, CHN	OP390450
CH-GXBS02-2023	2023	Baise, GX, CHN	PP460562	GXWZ-OP390452-2022	2022	Wuzhou, GX, CHN	OP390452
CH-GXBS03-2023	2023	Baise, GX, CHN	PP460563	CH-KT323979-1998	1998	CHN	KT323979
CH-GXBS04-2023	2023	Baise, GX, CHN	PP460564	CH-AY653204-2004	2004	CHN	AY653204
CH-GXBS05-2023	2023	Baise, GX, CHN	PP460565	CH-EF185992-2006	2006	CHN	EF185992
CH-GXBS06-2023	2023	Baise, GX, CHN	PP460566	CH-JN547228-2011	2011	CHN	JN547228
CH-GXBS07-2023	2023	Baise, GX, CHN	PP460567	CH-JN980698-2011	2011	CHN	JN980698
CH-GXBS08-2023	2023	Baise, GX, CHN	PP460568	CH-JX145339-2011	2011	CHN	JX145339
CH-GXBS09-2023	2023	Baise, GX, CHN	PP460569	CH-JX489155-2011	2011	CHN	JX489155
CH-GXBS10-2023	2023	Baise, GX, CHN	PP460570	CH-KC886306-2011	2011	CHN	KC886306
CH-GXGG01-2023	2023	Guigang, GX, CHN	PP460571	CH-KJ158152-2011	2011	CHN	KJ158152
CH-GXGG02-2023	2023	Guigang, GX, CHN	PP460572	CH-KM609204-2011	2011	CHN	KM609204
CH-GXGG03-2023	2023	Guigang, GX, CHN	PP460573	CH-JX112709-2012	2012	CHN	JX112709
CH-GXGG04-2023	2023	Guigang, GX, CHN	PP460574	CH-KF384500-2012	2012	CHN	KF384500
CH-GXLZ01-2023	2023	Liuzhou, GX, CHN	PP460575	CH-KM089829-2012	2012	CHN	KM089829
CH-GXNN01-2023	2023	Nanning, GX, CHN	PP460576	CH-KF601200-2013	2013	CHN	KF601200
CH-GXNN02-2023	2023	Nanning, GX, CHN	PP460577	CH-KF761675-2013	2013	CHN	KF761675

CH-GXNN03-2023	2023	Nanning, GX, CHN	PP460578	CH-KJ020932-2013	2013	CHN	KJ020932
CH-GXNN04-2023	2023	Nanning, GX, CHN	PP460579	CH-KJ646590-2013	2013	CHN	KJ646590
CH-GXNN05-2023	2023	Nanning, GX, CHN	PP460580	CH-KM609209-2013	2013	CHN	KM609209
CH-GXNN06-2023	2023	Nanning, GX, CHN	PP460581	CH-KM887144-2013	2013	CHN	KM887144
CH-GXNN07-2023	2023	Nanning, GX, CHN	PP460582	CH-KP765609-2013	2013	CHN	KP765609
CH-GXNN08-2023	2023	Nanning, GX, CHN	PP460583	CH-KR818832-2013	2013	CHN	KR818832
CH-GXNN09-2023	2023	Nanning, GX, CHN	PP460584	CH-KT021228-2013	2013	CHN	KT021228
CH-GXNN10-2023	2023	Nanning, GX, CHN	PP460585	CH-KU558701-2013	2013	CHN	KU558701
CH-GXNN11-2023	2023	Nanning, GX, CHN	PP460586	CH-KJ646580-2014	2014	CHN	KJ646580
CH-GXNN12-2023	2023	Nanning, GX, CHN	PP460587	CH-KM609212-2014	2014	CHN	KM609212
CH-GXNN13-2023	2023	Nanning, GX, CHN	PP460588	CH-KM609213-2014	2014	CHN	KM609213
CH-GXQZ01-2023	2023	Qinzhou, GX, CHN	PP460589	CH-KP276246-2014	2014	CHN	KP276246
CH-GXQZ02-2023	2023	Qinzhou, GX, CHN	PP460590	CH-KR153325-2014	2014	CHN	KR153325
CH-GXQZ03-2023	2023	Qinzhou, GX, CHN	PP460591	CH-KR296673-2014	2014	CHN	KR296673
CH-GXYL01-2023	2023	Yulin, GX, CHN	PP460592	CH-KT428879-2014	2014	CHN	KT428879
CH-GXGG01-2024	2024	Guigang, GX, CHN	PP460596	CH-KU133238-2014	2014	CHN	KU133238
CH-GXHZ01-2024	2024	Hezhou, GX, CHN	PP460593	CH-KX791060-2014	2014	CHN	KX791060
CH-GXHZ02-2024	2024	Hezhou, GX, CHN	PP460594	CH-KX016034-2014	2014	CHN	KX016034
CH-GXHZ03-2024	2024	Hezhou, GX, CHN	PP460595	CH-KR296664-2015	2015	CHN	KR296664
CH-GXLZ01-2024	2024	Liuzhou, GX, CHN	PP460597	CH-KR296667-2015	2015	CHN	KR296667
AU-KT206204-2015	2015	Austria	KT206204	CH-KR809885-2015	2015	CHN	KR809885
BEL-KR003452-2015	2015	Belgium	KR003452	CH-KU847996-2015	2015	CHN	KU847996
CAN-KM189367-2014	2014	Canada	KM189367	CH-KX534205-2015	2015	CHN	KX534205
CAN-KM196109-2014	2014	Canada	KM196109	CH-KY420075-2015	2015	CHN	KY420075
CAN-KR265831-2014	2014	Canada	KR265831	CH-KY793536-2015	2015	CHN	KY793536
COL-KU569509-2014	2014	Colombia	KU569509	CH-KY929405-2015	2015	CHN	KY929405

France-KR011756-2014	2014	France	KR011756	CH-MF152597-2015	2015	CHN	MF152597
GER-LM645058-2014	2014	Germany	LM645058	CH-MG742381-2015	2015	CHN	MG742381
JPN-LC063820-2013	2013	Japan	LC063820	CH-KY070587-2016	2016	CHN	KY070587
JPN-LC063836-2013	2013	Japan	LC063836	CH-KY211053-2016	2016	CHN	KY211053
KOR-JQ023161-2009	2009	Korea	JQ023161	CH-KY775049-2016	2016	CHN	KY775049
KOR-GU937797-2010	2010	Korea	GU937797	CH-MF346935-2016	2016	CHN	MF346935
KOR-JQ023162-2011	2011	Korea	JQ023162	CH-MF462814-2016	2016	CHN	MF462814
KOR-KR873431-2014	2014	Korea	KR873431	CH-MG020551-2016	2016	CHN	MG020551
KOR-OR529201-2022	2022	Korea	OR529201	CH-MG373533-2016	2016	CHN	MG373533
KOR-OR529205-2022	2022	Korea	OR529205	CH-MZ364310-2016	2016	CHN	MZ364310
KOR-PP107941-2022	2022	Korea	PP107941	CH-MH726407-2017	2017	CHN	MH726407
KOR-PP146394-2022	2022	Korea	PP146394	CH-MH061339-2017	2017	CHN	MH061339
MEX-KJ645708-2013	2013	Mexico	KJ645708	CH-MT547179-2017	2017	CHN	MT547179
NL-KR011122-2014	2014	Netherlands	KR011122	CH-MZ364311-2017	2017	CHN	MZ364311
THA-KR610991-2014	2014	Thailand	KR610991	CH-OR992088-2017	2017	CHN	OR992088
THA-LC053455-2015	2015	Thailand	LC053455	CH-MH061343-2018	2018	CHN	MH061343
UKR-KP403954-2014	2014	Ukraine	KP403954	CH-MH816969-2018	2018	CHN	MH816969
USA-KF272920-2013	2013	USA	KF272920	CH-MK135453-2018	2018	CHN	MK135453
USA-KF452323-2013	2013	USA	KF452323	CH-MZ364312-2018	2018	CHN	MZ364312
USA-KJ645642-2013	2013	USA	KJ645642	CH-MZ364313-2018	2018	CHN	MZ364313
USA-KJ645657-2013	2013	USA	KJ645657	CH-MZ364314-2018	2018	CHN	MZ364314
USA-KJ645695-2013	2013	USA	KJ645695	CH-MN368699-2019	2019	CHN	MN368699
USA-KR078299-2013	2013	USA	KR078299	CH-MZ364316-2019	2019	CHN	MZ364316
USA-KJ399978-2014	2014	USA	KJ399978	CH-ON988090-2019	2019	CHN	ON988090
USA-KR265811-2014	2014	USA	KR265811	CH-ON988085-2020	2020	CHN	ON988085
USA-KR265787-2014	2014	USA	KR265787	CH-ON988095-2020	2020	CHN	ON988095

USA-KY499262-2017	2017	USA	KY499262	CH-OQ269589-2020	2020	CHN	OQ269589
				CH-OR365279-2020	2020	CHN	OR365279
				CH-OR734225-2020	2020	CHN	OR734225
				CH-OP603897-2021	2021	CHN	OP603897
				CH-OQ122101-2021	2021	CHN	OQ122101
				CH-OQ122105-2021	2021	CHN	OQ122105
				CH-OQ504179-2021	2021	CHN	OQ504179
				CH-OR001818-2021	2021	CHN	OR001818
				CH-OR234022-2021	2021	CHN	OR234022
				CH-OR026663-2021	2021	CHN	OR026663
				CH-OR722805-2021	2021	CHN	OR722805
				CH-OM948926-2021	2021	CHN	OM948926
				CH-OR195033-2022	2022	CHN	OR195033
				CH-OR418363-2022	2022	CHN	OR418363
				CH-OR418364-2022	2022	CHN	OR418364
				CH-OR501721-2022	2022	CHN	OR501721
				CH-OR576824-2022	2022	CHN	OR576824
				CH-OR608353-2022	2022	CHN	OR608353
				CH-OR804100-2022	2022	CHN	OR804100
				CH-PP083729-2022	2022	CHN	PP083729
				CH-OR684450-2023	2023	CHN	OR684450
				CH-OR684451-2023	2023	CHN	OR684451
				CH-OR684452-2023	2023	CHN	OR684452
				CH-PP003006-2023	2023	CHN	PP003006

Note: GX: Guangxi province of China; CHN: China; JPN: Japan; KOR: Korea; USA: the United States of America; COL: Colombia; GER: Germany; NL: Netherlands; AU: Austria; CAN: Canada; BEL: Belgium; UKR: Ukraine; THA: Thailand; MEX: Mexico. The strains obtained in this study are marked in red. The same as follows.

Supplementary Table S2. The information on M gene of PEDV strains used in this study.

Virus Strain	Date	Origin	Accession No.	Virus Strain	Date	Origin	Accession No.
CH-GXBS01-2020	2020	Baise, GX, CHN	OR659272	Suisse-AF353511-2001	2001	Suisse	AF353511
CH-GXBS02-2020	2020	Baise, GX, CHN	OR659273	AJ1102-JX188454-2012	2012	CHN	JX188454
CH-GXBS03-2020	2020	Baise, GX, CHN	OR659274	CH-KT323979-1998	1998	CHN	KT323979
CH-GXBS01-2021	2021	Baise, GX, CHN	OR659275	CH-EF185992-2006	2006	CHN	EF185992
CH-GXCZ01-2021	2021	Chongzuo, GX, CHN	OR659292	CH-JN547228-2011	2011	CHN	JN547228
CH-GXCZ02-2021	2021	Chongzuo, GX, CHN	OR659293	CH-JX188454-2011	2011	CHN	JX188454
CH-GXCZ03-2021	2021	Chongzuo, GX, CHN	OR659294	CH-JX489155-2011	2011	CHN	JX489155
CH-GXCZ04-2021	2021	Chongzuo, GX, CHN	OR659295	CH-KC196276-2011	2011	CHN	KC196276
CH-GXCZ05-2021	2021	Chongzuo, GX, CHN	OR659296	CH-KJ158152-2011	2011	CHN	KJ158152
CH-GXCZ06-2021	2021	Chongzuo, GX, CHN	OR659297	CH-KM609204-2011	2011	CHN	KM609204
CH-GXNN01-2021	2021	Nanning, GX, CHN	OR659312	CH-JX112709-2012	2012	CHN	JX112709
CH-GXNN02-2021	2021	Nanning, GX, CHN	OR659313	CH-KF384500-2012	2012	CHN	KF384500
CH-GXBH01-2022	2022	Beihai, GX, CHN	OR659271	CH-KM089829-2012	2012	CHN	KM089829
CH-GXBS01-2022	2022	Baise, GX, CHN	OR659276	CH-KJ020932-2013	2013	CHN	KJ020932
CH-GXBS02-2022	2022	Baise, GX, CHN	OR659277	CH-KM609209-2013	2013	CHN	KM609209
CH-GXBS03-2022	2022	Baise, GX, CHN	OR659278	CH-KM887144-2013	2013	CHN	KM887144
CH-GXBS04-2022	2022	Baise, GX, CHN	OR659279	CH-KP765609-2013	2013	CHN	KP765609
CH-GXBS05-2022	2022	Baise, GX, CHN	OR659280	CH-KR818832-2013	2013	CHN	KR818832
CH-GXBS06-2022	2022	Baise, GX, CHN	OR659281	CH-KT021228-2013	2013	CHN	KT021228
CH-GXBS07-2022	2022	Baise, GX, CHN	OR659282	CH-KU558701-2013	2013	CHN	KU558701
CH-GXBS08-2022	2022	Baise, GX, CHN	OR659283	CH-KM609212-2014	2014	CHN	KM609212
CH-GXBS09-2022	2022	Baise, GX, CHN	OR659284	CH-KM609213-2014	2014	CHN	KM609213

CH-GXBS10-2022	2022	Baise, GX, CHN	OR659285	CH-KR153325-2014	2014	CHN	KR153325
CH-GXBS11-2022	2022	Baise, GX, CHN	OR659286	CH-KX016034-2014	2014	CHN	KX016034
CH-GXBS12-2022	2022	Baise, GX, CHN	OR659287	CH-KX791060-2014	2014	CHN	KX791060
CH-GXBS13-2022	2022	Baise, GX, CHN	OR659288	CH-KP890336-2015	2015	CHN	KP890336
CH-GXBS14-2022	2022	Baise, GX, CHN	OR659289	CH-KR095279-2015	2015	CHN	KR095279
CH-GXBS15-2022	2022	Baise, GX, CHN	OR659290	CH-KR809885-2015	2015	CHN	KR809885
CH-GXBS16-2022	2022	Baise, GX, CHN	OR659291	CH-KU847996-2015	2015	CHN	KU847996
CH-GXBS17-2022	2022	Baise, GX, CHN	PP460598	CH-KX534205-2015	2015	CHN	KX534205
CH-GXCZ01-2022	2022	Chongzuo, GX, CHN	OR659298	CH-KY420075-2015	2015	CHN	KY420075
CH-GXGG01-2022	2022	Guigang, GX, CHN	OR659299	CH-KY793536-2015	2015	CHN	KY793536
CH-GXGG02-2022	2022	Guigang, GX, CHN	OR659300	CH-KY929405-2015	2015	CHN	KY929405
CH-GXGG03-2022	2022	Guigang, GX, CHN	OR659301	CH-KY070587-2016	2016	CHN	KY070587
CH-GXGG04-2022	2022	Guigang, GX, CHN	OR659302	CH-MF346935-2016	2016	CHN	MF346935
CH-GXGG05-2022	2022	Guigang, GX, CHN	OR659303	CH-MF462814-2016	2016	CHN	MF462814
CH-GXGG06-2022	2022	Guigang, GX, CHN	OR659304	CH-MT547180-2016	2016	CHN	MT547180
CH-GXGG07-2022	2022	Guigang, GX, CHN	OR659305	CH-MZ364307-2016	2016	CHN	MZ364307
CH-GXGG08-2022	2022	Guigang, GX, CHN	OR659306	CH-MZ364308-2016	2016	CHN	MZ364308
CH-GXGG09-2022	2022	Guigang, GX, CHN	OR659307	CH-MZ364309-2016	2016	CHN	MZ364309
CH-GXGG10-2022	2022	Guigang, GX, CHN	OR659308	CH-MZ364310-2016	2016	CHN	MZ364310
CH-GXGG11-2022	2022	Guigang, GX, CHN	OR659309	CH-MH061338-2017	2017	CHN	MH061338
CH-GXGG12-2022	2022	Guigang, GX, CHN	OR659310	CH-MH061339-2017	2017	CHN	MH061339
CH-GXGG13-2022	2022	Guigang, GX, CHN	OR659311	CH-MH726407-2017	2017	CHN	MH726407
CH-GXLB01-2022	2022	Laibin, GX, CHN	PP460599	CH-MT547179-2017	2017	CHN	MT547179
CH-GXLZ01-2022	2022	Liuzhou, GX, CHN	PP460600	CH-MZ364311-2017	2017	CHN	MZ364311
CH-GXNN01-2022	2022	Nanning, GX, CHN	OR659314	CH-MH061343-2018	2018	CHN	MH061343
CH-GXNN02-2022	2022	Nanning, GX, CHN	OR659315	CH-MZ364312-2018	2018	CHN	MZ364312

CH-GXNN03-2022	2022	Nanning, GX, CHN	OR659316	CH-MZ364313-2018	2018	CHN	MZ364313
CH-GXNN04-2022	2022	Nanning, GX, CHN	OR659317	CH-MZ364314-2018	2018	CHN	MZ364314
CH-GXNN05-2022	2022	Nanning, GX, CHN	OR659318	CH-MZ364315-2019	2019	CHN	MZ364315
CH-GXNN06-2022	2022	Nanning, GX, CHN	OR659319	CH-MZ364316-2019	2019	CHN	MZ364316
CH-GXNN07-2022	2022	Nanning, GX, CHN	OR659320	CH-OQ269589-2020	2020	CHN	OQ269589
CH-GXNN08-2022	2022	Nanning, GX, CHN	OR659321	CH-OR734225-2020	2020	CHN	OR734225
CH-GXNN09-2022	2022	Nanning, GX, CHN	OR659322	CH-OR234022-2021	2021	CHN	OR234022
CH-GXBS01-2023	2023	Baise, GX, CHN	PP460601	CH-OR722805-2021	2021	CHN	OR722805
CH-GXBS02-2023	2023	Baise, GX, CHN	PP460602	CH-OR418363-2022	2022	CHN	OR418363
CH-GXBS03-2023	2023	Baise, GX, CHN	PP460603	CH-OR418364-2022	2022	CHN	OR418364
CH-GXBS04-2023	2023	Baise, GX, CHN	PP460604	CH-OR684450-2023	2023	CHN	OR684450
CH-GXBS05-2023	2023	Baise, GX, CHN	PP460605	CH-OR684451-2023	2023	CHN	OR684451
CH-GXBS06-2023	2023	Baise, GX, CHN	PP460606	Belgium-KR003452-2015	2015	Belgium	KR003452
CH-GXBS07-2023	2023	Baise, GX, CHN	PP460607	Canada-KR265831-2014	2014	Canada	KR265831
CH-GXBS08-2023	2023	Baise, GX, CHN	PP460608	Canada-KM189367-2014	2014	Canada	KM189367
CH-GXBS09-2023	2023	Baise, GX, CHN	PP460609	Colombia-KU569509-2014	2014	Colombia	KU569509
CH-GXBS10-2023	2023	Baise, GX, CHN	PP460610	France-KR011756-2014	2014	France	KR011756
CH-GXGG01-2023	2023	Guigang, GX, CHN	PP460611	Germany-LM645057-2014	2014	Germany	LM645057
CH-GXGG02-2023	2023	Guigang, GX, CHN	PP460612	Germany-LM645058-2014	2014	Germany	LM645058
CH-GXGG03-2023	2023	Guigang, GX, CHN	PP460613	JPN-LC063814-2013	2013	Japan	LC063814
CH-GXGG04-2023	2023	Guigang, GX, CHN	PP460614	JPN-LC063820-2013	2013	Japan	LC063820
CH-GXLZ01-2023	2023	Liuzhou, GX, CHN	PP460615	JPN-LC063836-2013	2013	Japan	LC063836
CH-GXNN01-2023	2023	Nanning, GX, CHN	PP460616	JPN-LC022792-2014	2014	Japan	LC022792
CH-GXNN02-2023	2023	Nanning, GX, CHN	PP460617	JPN-LC063822-2014	2014	Japan	LC063822
CH-GXNN03-2023	2023	Nanning, GX, CHN	PP460618	JPN-LC063828-2014	2014	Japan	LC063828
CH-GXNN04-2023	2023	Nanning, GX, CHN	PP460619	KOR-GU937797-2010	2010	Korea	GU937797

CH-GXNN05-2023	2023	Nanning, GX, CHN	PP460620	KOR-JQ023162-2011	2011	Korea	JQ023162
CH-GXNN06-2023	2023	Nanning, GX, CHN	PP460621	KOR-KR873431-2014	2014	Korea	KR873431
CH-GXNN07-2023	2023	Nanning, GX, CHN	PP460622	KOR-OK465397-2021	2021	Korea	OK465397
CH-GXNN08-2023	2023	Nanning, GX, CHN	PP460623	KOR-PP107941-2022	2022	Korea	PP107941
CH-GXNN09-2023	2023	Nanning, GX, CHN	PP460624	Mexico-KJ645708-2013	2013	Mexico	KJ645708
CH-GXNN10-2023	2023	Nanning, GX, CHN	PP460625	Thailand-KR610991-2014	2014	Thailand	KR610991
CH-GXNN11-2023	2023	Nanning, GX, CHN	PP460626	Ukraine-KP403954-2014	2014	Ukraine	KP403954
CH-GXNN12-2023	2023	Nanning, GX, CHN	PP460627	USA-KF272920-2013	2013	USA	KF272920
CH-GXNN13-2023	2023	Nanning, GX, CHN	PP460628	USA-KF452323-2013	2013	USA	KF452323
CH-GXQZ01-2023	2023	Qinzhou, GX, CHN	PP460629	USA-KR078299-2013	2013	USA	KR078299
CH-GXQZ02-2023	2023	Qinzhou, GX, CHN	PP460630	USA-KJ645642-2013	2013	USA	KJ645642
CH-GXQZ03-2023	2023	Qinzhou, GX, CHN	PP460631	USA-KJ645657-2013	2013	USA	KJ645657
CH-GXYL01-2023	2023	Yulin, GX, CHN	PP460632	USA-KJ645695-2013	2013	USA	KJ645695
CH-GXGG01-2024	2024	Guigang, GX, CHN	PP460636	USA-KR265811-2014	2014	USA	KR265811
CH-GXHZ01-2024	2024	Hezhou, GX, CHN	PP460633	USA-KJ265787-2014	2014	USA	KJ265787
CH-GXHZ02-2024	2024	Hezhou, GX, CHN	PP460634	USA-KJ399978-2014	2014	USA	KJ399978
CH-GXHZ03-2024	2024	Hezhou, GX, CHN	PP460635	USA-KY499262-2017	2017	USA	KY499262
CH-GXLZ01-2024	2024	Liuzhou, GX, CHN	PP460637				

Supplementary Table S3. The information on N gene of PEDV strains used in this study.

Virus Strain	Date	Origin	Accession No.	Virus Strain	Date	Origin	Accession No.
CH-GXBS01-2020	2020	Baise, GX, CHN	OR659324	Suisse-AF353511-2001	2001	Suisse	AF353511
CH-GXBS02-2020	2020	Baise, GX, CHN	OR659325	AJ1102-JX188454-2012	2012	CHN	JX188454
CH-GXBS03-2020	2020	Baise, GX, CHN	OR659326	CH-KT323979-1998	1998	CHN	KT323979
CH-GXBS01-2021	2021	Baise, GX, CHN	OR659327	CH-EF185992-2006	2006	CHN	EF185992
CH-GXCZ01-2021	2021	Chongzuo, GX, CHN	OR659344	CH-JN547228-2011	2011	CHN	JN547228
CH-GXCZ02-2021	2021	Chongzuo, GX, CHN	OR659345	CH-JX188454-2011	2011	CHN	JX188454
CH-GXCZ03-2021	2021	Chongzuo, GX, CHN	OR659346	CH-JX489155-2011	2011	CHN	JX489155
CH-GXCZ04-2021	2021	Chongzuo, GX, CHN	OR659347	CH-KC196276-2011	2011	CHN	KC196276
CH-GXCZ05-2021	2021	Chongzuo, GX, CHN	OR659348	CH-KJ158152-2011	2011	CHN	KJ158152
CH-GXCZ06-2021	2021	Chongzuo, GX, CHN	OR659349	CH-KM609204-2011	2011	CHN	KM609204
CH-GXNN01-2021	2021	Nanning, GX, CHN	OR659364	CH-JX112709-2012	2012	CHN	JX112709
CH-GXNN02-2021	2021	Nanning, GX, CHN	OR659365	CH-KF384500-2012	2012	CHN	KF384500
CH-GXBH01-2022	2022	Beihai, GX, CHN	OR659323	CH-KM089829-2012	2012	CHN	KM089829
CH-GXBS01-2022	2022	Baise, GX, CHN	OR659328	CH-KJ020932-2013	2013	CHN	KJ020932
CH-GXBS02-2022	2022	Baise, GX, CHN	OR659329	CH-KM609209-2013	2013	CHN	KM609209
CH-GXBS03-2022	2022	Baise, GX, CHN	OR659330	CH-KM887144-2013	2013	CHN	KM887144
CH-GXBS04-2022	2022	Baise, GX, CHN	OR659331	CH-KP765609-2013	2013	CHN	KP765609
CH-GXBS05-2022	2022	Baise, GX, CHN	OR659332	CH-KR818832-2013	2013	CHN	KR818832
CH-GXBS06-2022	2022	Baise, GX, CHN	OR659333	CH-KT021228-2013	2013	CHN	KT021228
CH-GXBS07-2022	2022	Baise, GX, CHN	OR659334	CH-KU558701-2013	2013	CHN	KU558701
CH-GXBS08-2022	2022	Baise, GX, CHN	OR659335	CH-KM609212-2014	2014	CHN	KM609212
CH-GXBS09-2022	2022	Baise, GX, CHN	OR659336	CH-KM609213-2014	2014	CHN	KM609213

CH-GXBS10-2022	2022	Baise, GX, CHN	OR659337	CH-KR153325-2014	2014	CHN	KR153325
CH-GXBS11-2022	2022	Baise, GX, CHN	OR659338	CH-KX016034-2014	2014	CHN	KX016034
CH-GXBS12-2022	2022	Baise, GX, CHN	OR659339	CH-KX791060-2014	2014	CHN	KX791060
CH-GXBS13-2022	2022	Baise, GX, CHN	OR659340	CH-KP890336-2015	2015	CHN	KP890336
CH-GXBS14-2022	2022	Baise, GX, CHN	OR659341	CH-KR095279-2015	2015	CHN	KR095279
CH-GXBS15-2022	2022	Baise, GX, CHN	OR659342	CH-KR809885-2015	2015	CHN	KR809885
CH-GXBS16-2022	2022	Baise, GX, CHN	OR659343	CH-KU847996-2015	2015	CHN	KU847996
CH-GXBS17-2022	2022	Baise, GX, CHN	PP460638	CH-KX534205-2015	2015	CHN	KX534205
CH-GXCZ01-2022	2022	Chongzuo, GX, CHN	OR659350	CH-KY420075-2015	2015	CHN	KY420075
CH-GXGG01-2022	2022	Guigang, GX, CHN	OR659351	CH-KY793536-2015	2015	CHN	KY793536
CH-GXGG02-2022	2022	Guigang, GX, CHN	OR659352	CH-KY929405-2015	2015	CHN	KY929405
CH-GXGG03-2022	2022	Guigang, GX, CHN	OR659353	CH-KY070587-2016	2016	CHN	KY070587
CH-GXGG04-2022	2022	Guigang, GX, CHN	OR659354	CH-MF346935-2016	2016	CHN	MF346935
CH-GXGG05-2022	2022	Guigang, GX, CHN	OR659355	CH-MF462814-2016	2016	CHN	MF462814
CH-GXGG06-2022	2022	Guigang, GX, CHN	OR659356	CH-MT547180-2016	2016	CHN	MT547180
CH-GXGG07-2022	2022	Guigang, GX, CHN	OR659357	CH-MZ364307-2016	2016	CHN	MZ364307
CH-GXGG08-2022	2022	Guigang, GX, CHN	OR659358	CH-MZ364308-2016	2016	CHN	MZ364308
CH-GXGG09-2022	2022	Guigang, GX, CHN	OR659359	CH-MZ364309-2016	2016	CHN	MZ364309
CH-GXGG10-2022	2022	Guigang, GX, CHN	OR659360	CH-MZ364310-2016	2016	CHN	MZ364310
CH-GXGG11-2022	2022	Guigang, GX, CHN	OR659361	CH-MH061338-2017	2017	CHN	MH061338
CH-GXGG12-2022	2022	Guigang, GX, CHN	OR659362	CH-MH061339-2017	2017	CHN	MH061339
CH-GXGG13-2022	2022	Guigang, GX, CHN	OR659363	CH-MH726407-2017	2017	CHN	MH726407
CH-GXLB01-2022	2022	Laibin, GX, CHN	PP460639	CH-MT547179-2017	2017	CHN	MT547179
CH-GXLZ01-2022	2022	Liuzhou, GX, CHN	PP460640	CH-MZ364311-2017	2017	CHN	MZ364311
CH-GXNN01-2022	2022	Nanning, GX, CHN	OR659366	CH-MH061343-2018	2018	CHN	MH061343
CH-GXNN02-2022	2022	Nanning, GX, CHN	OR659367	CH-MZ364312-2018	2018	CHN	MZ364312

CH-GXNN03-2022	2022	Nanning, GX, CHN	OR659368	CH-MZ364313-2018	2018	CHN	MZ364313
CH-GXNN04-2022	2022	Nanning, GX, CHN	OR659369	CH-MZ364314-2018	2018	CHN	MZ364314
CH-GXNN05-2022	2022	Nanning, GX, CHN	OR659370	CH-MZ364315-2019	2019	CHN	MZ364315
CH-GXNN06-2022	2022	Nanning, GX, CHN	OR659371	CH-MZ364316-2019	2019	CHN	MZ364316
CH-GXNN07-2022	2022	Nanning, GX, CHN	OR659372	CH-OQ269589-2020	2020	CHN	OQ269589
CH-GXNN08-2022	2022	Nanning, GX, CHN	OR659373	CH-OR734225-2020	2020	CHN	OR734225
CH-GXNN09-2022	2022	Nanning, GX, CHN	OR659374	CH-OR234022-2021	2021	CHN	OR234022
CH-GXBS01-2023	2023	Baise, GX, CHN	PP460641	CH-OR722805-2021	2021	CHN	OR722805
CH-GXBS02-2023	2023	Baise, GX, CHN	PP460642	CH-OR418363-2022	2022	CHN	OR418363
CH-GXBS03-2023	2023	Baise, GX, CHN	PP460643	CH-OR418364-2022	2022	CHN	OR418364
CH-GXBS04-2023	2023	Baise, GX, CHN	PP460644	CH-OR684450-2023	2023	CHN	OR684450
CH-GXBS05-2023	2023	Baise, GX, CHN	PP460645	CH-OR684451-2023	2023	CHN	OR684451
CH-GXBS06-2023	2023	Baise, GX, CHN	PP460646	Belgium-KR003452-2015	2015	Belgium	KR003452
CH-GXBS07-2023	2023	Baise, GX, CHN	PP460647	Canada-KR265831-2014	2014	Canada	KR265831
CH-GXBS08-2023	2023	Baise, GX, CHN	PP460648	Canada-KM189367-2014	2014	Canada	KM189367
CH-GXBS09-2023	2023	Baise, GX, CHN	PP460649	Colombia-KU569509-2014	2014	Colombia	KU569509
CH-GXBS10-2023	2023	Baise, GX, CHN	PP460650	France-KR011756-2014	2014	France	KR011756
CH-GXGG01-2023	2023	Guigang, GX, CHN	PP460651	Germany-LM645057-2014	2014	Germany	LM645057
CH-GXGG02-2023	2023	Guigang, GX, CHN	PP460652	Germany-LM645058-2014	2014	Germany	LM645058
CH-GXGG03-2023	2023	Guigang, GX, CHN	PP460653	JPN-LC063814-2013	2013	Japan	LC063814
CH-GXGG04-2023	2023	Guigang, GX, CHN	PP460654	JPN-LC063820-2013	2013	Japan	LC063820
CH-GXLZ01-2023	2023	Liuzhou, GX, CHN	PP460655	JPN-LC063836-2013	2013	Japan	LC063836
CH-GXNN01-2023	2023	Nanning, GX, CHN	PP460656	JPN-LC022792-2014	2014	Japan	LC022792
CH-GXNN02-2023	2023	Nanning, GX, CHN	PP460657	JPN-LC063822-2014	2014	Japan	LC063822
CH-GXNN03-2023	2023	Nanning, GX, CHN	PP460658	JPN-LC063828-2014	2014	Japan	LC063828
CH-GXNN04-2023	2023	Nanning, GX, CHN	PP460659	KOR-GU937797-2010	2010	Korea	GU937797

CH-GXNN05-2023	2023	Nanning, GX, CHN	PP460660	KOR-JQ023162-2011	2011	Korea	JQ023162
CH-GXNN06-2023	2023	Nanning, GX, CHN	PP460661	KOR-KR873431-2014	2014	Korea	KR873431
CH-GXNN07-2023	2023	Nanning, GX, CHN	PP460662	KOR-OK465397-2021	2021	Korea	OK465397
CH-GXNN08-2023	2023	Nanning, GX, CHN	PP460663	KOR-PP107941-2022	2022	Korea	PP107941
CH-GXNN09-2023	2023	Nanning, GX, CHN	PP460664	Mexico-KJ645708-2013	2013	Mexico	KJ645708
CH-GXNN10-2023	2023	Nanning, GX, CHN	PP460665	Thailand-KR610991-2014	2014	Thailand	KR610991
CH-GXNN11-2023	2023	Nanning, GX, CHN	PP460666	Ukraine-KP403954-2014	2014	Ukraine	KP403954
CH-GXNN12-2023	2023	Nanning, GX, CHN	PP460667	USA-KF272920-2013	2013	USA	KF272920
CH-GXNN13-2023	2023	Nanning, GX, CHN	PP460668	USA-KF452323-2013	2013	USA	KF452323
CH-GXQZ01-2023	2023	Qinzhou, GX, CHN	PP460669	USA-KR078299-2013	2013	USA	KR078299
CH-GXQZ02-2023	2023	Qinzhou, GX, CHN	PP460670	USA-KJ645642-2013	2013	USA	KJ645642
CH-GXQZ03-2023	2023	Qinzhou, GX, CHN	PP460671	USA-KJ645657-2013	2013	USA	KJ645657
CH-GXYL01-2023	2023	Yulin, GX, CHN	PP460672	USA-KJ645695-2013	2013	USA	KJ645695
CH-GXGG01-2024	2024	Guigang, GX, CHN	PP460676	USA-KR265811-2014	2014	USA	KR265811
CH-GXHZ01-2024	2024	Hezhou, GX, CHN	PP460673	USA-KJ265787-2014	2014	USA	KJ265787
CH-GXHZ02-2024	2024	Hezhou, GX, CHN	PP460674	USA-KJ399978-2014	2014	USA	KJ399978
CH-GXHZ03-2024	2024	Hezhou, GX, CHN	PP460675	USA-KY499262-2017	2017	USA	KY499262
CH-GXLZ01-2024	2024	Liuzhou, GX, CHN	PP460677				

Supplementary Table S4. The information on S1 gene of PEDV strains from Guangxi province used in this study.

Virus Strain	Date	Origin	Accession No.	Virus Strain	Date	Origin	Accession No.
CH-GXBS01-2020	2020	Baise, GX, CHN	OR659220	CV777-AF353511-2001	2001	Suisse	AF353511
CH-GXBS02-2020	2020	Baise, GX, CHN	OR659221	AJ1102-JX188454-2012	2012	CHN	JX188454
CH-GXBS03-2020	2020	Baise, GX, CHN	OR659222	GX-JQ979288-2011	2011	GX, CHN	JQ979288
CH-GXBS01-2021	2021	Baise, GX, CHN	OR659223	GX-KY793536-2015	2015	GX, CHN	KY793536
CH-GXCZ01-2021	2021	Chongzuo, GX, CHN	OR659240	GX-MZ364307-2016	2016	GX, CHN	MZ364307
CH-GXCZ02-2021	2021	Chongzuo, GX, CHN	OR659241	GX-MZ364308-2016	2016	GX, CHN	MZ364308
CH-GXCZ03-2021	2021	Chongzuo, GX, CHN	OR659242	GX-MZ364309-2016	2016	GX, CHN	MZ364309
CH-GXCZ04-2021	2021	Chongzuo, GX, CHN	OR659243	GX-MZ364310-2016	2016	GX, CHN	MZ364310
CH-GXCZ05-2021	2021	Chongzuo, GX, CHN	OR659244	GXCZ-MK000562-2017	2017	Chongzuo, GX, CHN	MK000562
CH-GXCZ06-2021	2021	Chongzuo, GX, CHN	OR659245	GXCZ-MK000564-2017	2017	Chongzuo, GX, CHN	MK000564
CH-GXNN01-2021	2021	Nanning, GX, CHN	OR659260	GXGG-MK000570-2017	2017	Guigang, GX, CHN	MK000570
CH-GXNN02-2021	2021	Nanning, GX, CHN	OR659261	GXGG-MK000574-2017	2017	Guigang, GX, CHN	MK000574
CH-GXBH01-2022	2022	Beihai, GX, CHN	OR659219	GXGG-MK000575-2017	2017	Guigang, GX, CHN	MK000575
CH-GXBS01-2022	2022	Baise, GX, CHN	OR659224	GXGG-MK000576-2017	2017	Guigang, GX, CHN	MK000576
CH-GXBS02-2022	2022	Baise, GX, CHN	OR659225	GXGG-MK000577-2017	2017	Guigang, GX, CHN	MK000577
CH-GXBS03-2022	2022	Baise, GX, CHN	OR659226	GXGG-MK000582-2017	2017	Guigang, GX, CHN	MK000582
CH-GXBS04-2022	2022	Baise, GX, CHN	OR659227	GXNN-MK000578-2017	2017	Nanning, GX, CHN	MK000578
CH-GXBS05-2022	2022	Baise, GX, CHN	OR659228	GXNN-MK000563-2017	2017	Nanning, GX, CHN	MK000563
CH-GXBS06-2022	2022	Baise, GX, CHN	OR659229	GXQZ-MH985745-2017	2017	Qinzhou, GX, CHN	MH985745
CH-GXBS07-2022	2022	Baise, GX, CHN	OR659230	GXQZ-MK000566-2017	2017	Qinzhou, GX, CHN	MK000566
CH-GXBS08-2022	2022	Baise, GX, CHN	OR659231	GXQZ-MK000569-2017	2017	Qinzhou, GX, CHN	MK000569

CH-GXBS09-2022	2022	Baise, GX, CHN	OR659232	GXBS-MK731903-2017	2017	Baise, GX, CHN	MK731903
CH-GXBS10-2022	2022	Baise, GX, CHN	OR659233	GXBS-MK731904-2017	2017	Baise, GX, CHN	MK731904
CH-GXBS11-2022	2022	Baise, GX, CHN	OR659234	GXBS-MK731905-2017	2017	Baise, GX, CHN	MK731905
CH-GXBS12-2022	2022	Baise, GX, CHN	OR659235	GXCZ-MT547179-2017	2017	Chongzuo, GX, CHN	MT547179
CH-GXBS13-2022	2022	Baise, GX, CHN	OR659236	GXCZ-MT547180-2017	2017	Chongzuo, GX, CHN	MT547180
CH-GXBS14-2022	2022	Baise, GX, CHN	OR659237	GXGG-MN721373-2017	2017	Guigang, GX, CHN	MN721373
CH-GXBS15-2022	2022	Baise, GX, CHN	OR659238	GXHC-MK731906-2017	2017	Hechi, GX, CHN	MK731906
CH-GXBS16-2022	2022	Baise, GX, CHN	OR659239	GXLZ-MZ669841-2017	2017	Liuzhou, GX, CHN	MZ669841
CH-GXBS17-2022	2022	Baise, GX, CHN	PP460558	GXLZ-MZ669842-2017	2017	Liuzhou, GX, CHN	MZ669842
CH-GXCZ01-2022	2022	Chongzuo, GX, CHN	OR659246	GXLZ-MZ669843-2017	2017	Liuzhou, GX, CHN	MZ669843
CH-GXGG01-2022	2022	Guigang, GX, CHN	OR659247	GX-MK000563-2017	2017	GX, CHN	MK000563
CH-GXGG02-2022	2022	Guigang, GX, CHN	OR659248	GX-MZ364311-2017	2017	GX, CHN	MZ364311
CH-GXGG03-2022	2022	Guigang, GX,CHN	OR659249	GXBH-MK000572-2018	2018	Beihai,GX,CHN	MK000572
CH-GXGG04-2022	2022	Guigang,GX, CHN	OR659250	GXBH-MK000573-2018	2018	Beihai, GX, CHN	MK000573
CH-GXGG05-2022	2022	Guigang, GX, CHN	OR659251	GXBH-MK478139-2018	2018	Beihai, GX, CHN	MK478139
CH-GXGG06-2022	2022	Guigang, GX, CHN	OR659252	GXBH-MK478140-2018	2018	Beihai, GX, CHN	MK478140
CH-GXGG07-2022	2022	Guigang, GX, CHN	OR659253	GXBH-MK478146-2018	2018	Beihai, GX, CHN	MK478146
CH-GXGG08-2022	2022	Guigang,GX,CHN	OR659254	GXBH-MK478147-2018	2018	Beihai, GX, CHN	MK478147
CH-GXGG09-2022	2022	Guigang, GX, CHN	OR659255	GXBH-MK478148-2018	2018	Beihai, GX, CHN	MK478148
CH-GXGG10-2022	2022	Guigang, GX, CHN	OR659256	GXCZ-MK000571-2018	2018	Chongzuo, GX, CHN	MK000571
CH-GXGG11-2022	2022	Guigang, GX, CHN	OR659257	GXGG-MK000579-2018	2018	Guigang, GX, CHN	MK000579
CH-GXGG12-2022	2022	Guigang, GX, CHN	OR659258	GXGG-MK000580-2018	2018	Guigang, GX, CHN	MK000580
CH-GXGG13-2022	2022	Guigang, GX, CHN	OR659259	GXGG-MK000583-2018	2018	Guigang, GX, CHN	MK000583
CH-GXLB01-2022	2022	Laibin, GX, CHN	PP460559	GXGG-MK478145-2018	2018	Guigang, GX, CHN	MK478145
CH-GXLZ01-2022	2022	Liuzhou, GX, CHN	PP460560	GXGG-MK478150-2018	2018	Guigang, GX, CHN	MK478150
CH-GXNN01-2022	2022	Nanning, GX, CHN	OR659262	GXGG-MK478153-2018	2018	Guigang, GX, CHN	MK478153

CH-GXNN02-2022	2022	Nanning, GX, CHN	OR659263	GXGG-MK478154-2018	2018	Guigang, GX, CHN	MK478154
CH-GXNN03-2022	2022	Nanning, GX, CHN	OR659264	GXLB-MK478152-2018	2018	Laibin, GX, CHN	MK478152
CH-GXNN04-2022	2022	Nanning, GX, CHN	OR659265	GXLZ-MK478138-2018	2018	Liuzhou, GX, CHN	MK478138
CH-GXNN05-2022	2022	Nanning, GX, CHN	OR659266	GXNN-MK478136-2018	2018	Nanning, GX, CHN	MK478136
CH-GXNN06-2022	2022	Nanning, GX, CHN	OR659267	GXNN-MK478137-2018	2018	Nanning, GX, CHN	MK478137
CH-GXNN07-2022	2022	Nanning, GX, CHN	OR659268	GXNN-MK478141-2018	2018	Nanning, GX, CHN	MK478141
CH-GXNN08-2022	2022	Nanning, GX, CHN	OR659269	GXNN-MK478142-2018	2018	Nanning, GX, CHN	MK478142
CH-GXNN09-2022	2022	Nanning, GX, CHN	OR659270	GXNN-MK478149-2018	2018	Nanning, GX, CHN	MK478149
CH-GXBS01-2023	2023	Baise, GX, CHN	PP460561	GXNN-MK478151-2018	2018	Nanning, GX, CHN	MK478151
CH-GXBS02-2023	2023	Baise, GX, CHN	PP460562	GXNN-MK478155-2018	2018	Nanning, GX, CHN	MK478155
CH-GXBS03-2023	2023	Baise, GX, CHN	PP460563	GXQZ-MK478135-2018	2018	Qinzhou, GX, CHN	MK478135
CH-GXBS04-2023	2023	Baise, GX, CHN	PP460564	GXQZ-MK478143-2018	2018	Qinzhou, GX, CHN	MK478143
CH-GXBS05-2023	2023	Baise, GX, CHN	PP460565	GXQZ-MK478144-2018	2018	Qinzhou, GX, CHN	MK478144
CH-GXBS06-2023	2023	Baise, GX, CHN	PP460566	GXQZ-MN019125-2018	2018	Qinzhou, GX, CHN	MN019125
CH-GXBS07-2023	2023	Baise, GX, CHN	PP460567	GXQZ-MK000567-2018	2018	Qinzhou, GX, CHN	MK000567
CH-GXBS08-2023	2023	Baise, GX, CHN	PP460568	GXQZ-MK000568-2018	2018	Qinzhou, GX, CHN	MK000568
CH-GXBS09-2023	2023	Baise, GX, CHN	PP460569	GXYL-MK000561-2018	2018	YuLin, GX, CHN	MK000581
CH-GXBS10-2023	2023	Baise, GX, CHN	PP460570	GXYL-MK000564-2018	2018	YuLin, GX, CHN	MK000584
CH-GXGG01-2023	2023	Guigang, GX, CHN	PP460571	GXYL-MK000565-2018	2018	YuLin, GX, CHN	MK000565
CH-GXGG02-2023	2023	Guigang, GX, CHN	PP460572	GXBH-MK731907-2018	2018	Beihai, GX, CHN	MK731907
CH-GXGG03-2023	2023	Guigang, GX, CHN	PP460573	GXBH-MK731908-2018	2018	Beihai, GX, CHN	MK731908
CH-GXGG04-2023	2023	Guigang, GX, CHN	PP460574	GXBH-MK731909-2018	2018	Beihai, GX, CHN	MK731909
CH-GXLZ01-2023	2023	Liuzhou, GX, CHN	PP460575	GXBH-MZ669844-2018	2018	Beihai, GX, CHN	MZ669844
CH-GXNN01-2023	2023	Nanning, GX, CHN	PP460576	GXBH-MZ669845-2018	2018	Beihai, GX, CHN	MZ669845
CH-GXNN02-2023	2023	Nanning, GX, CHN	PP460577	GXBS-MK731910-2018	2018	Baise, GX, CHN	MK731910
CH-GXNN03-2023	2023	Nanning, GX, CHN	PP460578	GXBS-MK731911-2018	2018	Baise, GX, CHN	MK731911

CH-GXNN04-2023	2023	Nanning, GX, CHN	PP460579	GXBS-MK731912-2018	2018	Baise, GX, CHN	MK731912
CH-GXNN05-2023	2023	Nanning, GX, CHN	PP460580	GXGG-MK731913-2018	2018	Guigang, GX, CHN	MK731913
CH-GXNN06-2023	2023	Nanning, GX, CHN	PP460581	GXGG-MK731914-2018	2018	Guigang, GX, CHN	MK731914
CH-GXNN07-2023	2023	Nanning, GX, CHN	PP460582	GXGG-MK731915-2018	2018	Guigang, GX, CHN	MK731915
CH-GXNN08-2023	2023	Nanning, GX, CHN	PP460583	GXGG-MK731916-2018	2018	Guigang, GX, CHN	MK731916
CH-GXNN09-2023	2023	Nanning, GX, CHN	PP460584	GXGG-MK731917-2018	2018	Guigang, GX, CHN	MK731917
CH-GXNN10-2023	2023	Nanning, GX, CHN	PP460585	GXGG-MK731918-2018	2018	Guigang, GX, CHN	MK731918
CH-GXNN11-2023	2023	Nanning, GX, CHN	PP460586	GXGG-MK731919-2018	2018	Guigang, GX, CHN	MK731919
CH-GXNN12-2023	2023	Nanning, GX, CHN	PP460587	GXGG-MK731920-2018	2018	Guigang, GX, CHN	MK731920
CH-GXNN13-2023	2023	Nanning, GX, CHN	PP460588	GXHZ-MK135453-2018	2018	Hechi, GX, CHN	MK135453
CH-GXQZ01-2023	2023	Qinzhou, GX, CHN	PP460589	GXLZ-MZ669843-2018	2018	Liuzhou, GX, CHN	MZ669843
CH-GXQZ02-2023	2023	Qinzhou, GX, CHN	PP460590	GX-MK731921-2018	2018	GX, CHN	MK731921
CH-GXQZ03-2023	2023	Qinzhou, GX, CHN	PP460591	GX-MK731922-2018	2018	GX, CHN	MK731922
CH-GXYL01-2023	2023	Yulin, GX, CHN	PP460592	GX-MZ364312-2018	2018	GX, CHN	MZ364312
CH-GXGG01-2024	2024	Guigang, GX, CHN	PP460596	GX-MZ364313-2018	2018	GX, CHN	MZ364313
CH-GXHZ01-2024	2024	Hezhou, GX, CHN	PP460593	GX-MZ364314-2018	2018	GX, CHN	MZ364314
CH-GXHZ02-2024	2024	Hezhou, GX, CHN	PP460594	GXNN-MZ669834-2018	2018	Nanning, GX, CHN	MZ669834
CH-GXHZ03-2024	2024	Hezhou, GX, CHN	PP460595	GXNN-MZ669835-2018	2018	Nanning, GX, CHN	MZ669835
CH-GXLZ01-2024	2024	Liuzhou, GX, CHN	PP460597	GXNN-MZ669836-2018	2018	Nanning, GX, CHN	MZ669836
Austria-KT206204-2015	2015	Austria	KT206204	GXNN-MZ669837-2018	2018	Nanning, GX, CHN	MZ669837
Belgium-KR003452-2015	2015	Belgium	KR003452	GXNN-MZ669838-2018	2018	Nanning, GX, CHN	MZ669838
Canada-KM189367-2014	2014	Canada	KM189367	GXNN-MZ669839-2018	2018	Nanning, GX, CHN	MZ669839
Colombia-KU569509-2014	2014	Colombia	KU569509	GXNN-MZ669840-2018	2018	Nanning, GX, CHN	MZ669840
France-KR011756-2014	2014	France	KR011756	GXYL-MK731923-2018	2018	Yulin, GX, CHN	MK731923
Germany-LM645058-2014	2014	Germany	LM645058	GXBH-MW114971-2019	2019	Beihai, GX, CHN	MW114971
JPN-LC062836-2013	2013	Japan	LC062836	GXBH-MW114972-2019	2019	Beihai, GX, CHN	MW114972

KOR-GU937797-2010	2010	Korea	GU937797
KOR-JQ023162-2011	2011	Korea	JQ023162
KOR-PP107941-2022	2022	Korea	PP107941
Mexico-KJ645708-2013	2013	Mexico	KJ645708
Netherlands-KR011122-2014	2014	Netherlands	KR011122
Ukraine-KP403954-2014	2014	Ukraine	KP403954
USA-KJ645695-2013	2013	USA	KJ645695
USA-KR078299-2013	2013	USA	KR078299
Thailand-LC053455-2015	2015	Thailand	LC053455
CH-KT323979-1998	1998	CHN	KT323979
CH-AY653204-2004	2004	CHN	AY653204
CH-EF185992-2006	2006	CHN	EF185992
CH-KT021228-2013	2013	CHN	KT021228
CH-KJ646580-2014	2014	CHN	KJ646580
CH-KU847996-2015	2015	CHN	KU847996
CH-KY420075-2015	2015	CHN	KY420075
CH-MK135453-2018	2018	CHN	MK135453
CH-OP603897-2021	2021	CHN	OP603897
CH-OR684452-2023	2023	CHN	OR684452
CH-PP003006-2023	2023	CHN	PP003006
GXBH-MW703493-2019	2019	Beihai, GX, CHN	MW703493
GXQZ-MN019126-2019	2019	Qinzhou, GX, CHN	MN019126
GXBH-MK731924-2019	2019	Beihai, GX, CHN	MK731924
GXBH-MK731925-2019	2019	Beihai, GX, CHN	MK731925
GXCZ-OR365272-2019	2019	Chongzuo, GX, CHN	OR365272
GX-MZ364315-2019	2019	GX, CHN	MZ364315
GX-MZ364316-2019	2019	GX, CHN	MZ364316
GXBH-MW114973-2020	2020	Beihai, GX, CHN	MW114973
GXNN-MW703496-2020	2020	Nanning, GX, CHN	MW703496
GXNN-MW703495-2020	2020	Nanning, GX, CHN	MW703495
GXNN-MW703497-2020	2020	Nanning, GX, CHN	MW703497
GXNN-MW703498-2020	2020	Nanning, GX, CHN	MW703498
GXNN-OP390411-2020	2020	Nanning, GX, CHN	OP390411
GXNN-OP390412-2020	2020	Nanning, GX, CHN	OP390412
GXGG-OP390410-2020	2020	Guigang, GX, CHN	OP390410
GXWZ-MW703494-2020	2020	Wuzhou, GX, CHN	MW703494
GXQZ-OP390413-2020	2020	Qinzhou, GX, CHN	OP390413
GXYL-OP390414-2020	2020	YuLin, GX, CHN	OP390414
GXBH-OP390415-2020	2020	Beihai, GX, CHN	OP390415
GXCZ-OR365273-2020	2020	Chongzuo, GX, CHN	OR365273
GXCZ-OR365274-2020	2020	Chongzuo, GX, CHN	OR365274
GXCZ-OR365275-2020	2020	Chongzuo, GX, CHN	OR365275
GXCZ-OR365276-2020	2020	Chongzuo, GX, CHN	OR365276
GXCZ-OR365277-2020	2020	Chongzuo, GX, CHN	OR365277
GXCZ-OR365278-2020	2020	Chongzuo, GX, CHN	OR365278
GXCZ-OR365279-2020	2020	Chongzuo, GX, CHN	OR365279

GXNN-MW762534-2020	2020	Nanning, GX, CHN	MW762534
GXNN-MZ703013-2020	2020	Nanning, GX, CHN	MZ703013
GXNN-MZ703014-2020	2020	Nanning, GX, CHN	MZ703014
GX-OM948907-2020	2020	GX, CHN	OM948907
GXBH-OP390416-2021	2021	Beihai, GX, CHN	OP390416
GXBH-OP390417-2021	2021	Beihai, GX, CHN	OP390417
GXBH-OP390418-2021	2021	Beihai, GX, CHN	OP390418
GXGG-OP390431-2021	2021	Guigang, GX, CHN	OP390431
GXGG-OP390438-2021	2021	Guigang, GX, CHN	OP390438
GXGG-OP390439-2021	2021	Guigang, GX, CHN	OP390439
GXGG-OP390443-2021	2021	Guigang, GX, CHN	OP390443
GXLB-OP390440-2021	2021	Laibin, GX, CHN	OP390440
GXLB-OP390441-2021	2021	Laibin, GX, CHN	OP390441
GXLB-OP390444-2021	2021	Laibin, GX, CHN	OP390444
GXLZ-OP390423-2021	2021	Liuzhou, GX, CHN	OP390423
GXLZ-OP390424-2021	2021	Liuzhou, GX, CHN	OP390424
GXLZ-OP390425-2021	2021	Liuzhou, GX, CHN	OP390425
GXLZ-OP390430-2021	2021	Liuzhou, GX, CHN	OP390430
GXLZ-OP390432-2021	2021	Liuzhou, GX, CHN	OP390432
GXLZ-OP390433-2021	2021	Liuzhou, GX, CHN	OP390433
GXLZ-OP390434-2021	2021	Liuzhou, GX, CHN	OP390434
GXLZ-OP390447-2021	2021	Liuzhou, GX, CHN	OP390447
GXLZ-OP390448-2021	2021	Liuzhou, GX, CHN	OP390448
GXNN-OP390419-2021	2021	Nanning, GX, CHN	OP390419
GXNN-OP390426-2021	2021	Nanning, GX, CHN	OP390426
GXNN-OP390428-2021	2021	Nanning, GX, CHN	OP390428

GXNN-OP390435-2021	2021	Nanning, GX, CHN	OP390435
GXNN-OP390436-2021	2021	Nanning, GX, CHN	OP390436
GXNN-OP390437-2021	2021	Nanning, GX, CHN	OP390437
GXNN-OP390442-2021	2021	Nanning, GX, CHN	OP390442
GXNN-OP390453-2021	2021	Nanning, GX, CHN	OP390453
GXQZ-OP390445-2021	2021	Qinzhou, GX, CHN	OP390445
GXWZ-OP390420-2021	2021	Wuzhou, GX, CHN	OP390420
GXWZ-OP390421-2021	2021	Wuzhou, GX, CHN	OP390421
GXWZ-OP390422-2021	2021	Wuzhou, GX, CHN	OP390422
GXWZ-OP390429-2021	2021	Wuzhou, GX, CHN	OP390429
GXBH-MZ703033-2021	2021	Beihai, GX, CHN	MZ703033
GXFCG-MZ703034-2021	2021	Fangchenggang, GX, CHN	MZ703034
GXGG-MZ703016-2021	2021	Guigang, GX, CHN	MZ703016
GXGG-MZ703017-2021	2021	Guigang, GX, CHN	MZ703017
GXGG-MZ703021-2021	2021	Guigang, GX, CHN	MZ703021
GXGG-MZ703022-2021	2021	Guigang, GX, CHN	MZ703022
GXGG-MZ703023-2021	2021	Guigang, GX, CHN	MZ703023
GXGG-MZ703027-2021	2021	Guigang, GX, CHN	MZ703027
GXGG-MZ703036-2021	2021	Guigang, GX, CHN	MZ703036
GXNN-MZ703018-2021	2021	Nanning, GX, CHN	MZ703018
GXNN-MZ703020-2021	2021	Nanning, GX, CHN	MZ703020
GXNN-MZ703028-2021	2021	Nanning, GX, CHN	MZ703028
GXNN-MZ703029-2021	2021	Nanning, GX, CHN	MZ703029
GXNN-MZ703030-2021	2021	Nanning, GX, CHN	MZ703030
GXNN-MZ703031-2021	2021	Nanning, GX, CHN	MZ703031
GXNN-MZ703035-2021	2021	Nanning, GX, CHN	MZ703035

GXNN-MZ703015-2021	2021	Nanning, GX, CHN	MZ703015
GXNN-OM948908-2021	2021	Nanning, GX, CHN	OM948908
GXQZ-MZ703032-2021	2021	Qinzhou, GX, CHN	MZ703032
GXWZ-MZ703024-2021	2021	Wuzhou, GX, CHN	MZ703024
GXWZ-MZ703037-2021	2021	Wuzhou, GX, CHN	MZ703037
GXWZ-MZ703038-2021	2021	Wuzhou, GX, CHN	MZ703038
GXYL-MZ703025-2021	2021	Yulin, GX, CHN	MZ703025
GXNN-OP390449-2022	2022	Nanning, GX, CHN	OP390449
GXNN-OP390450-2022	2022	Nanning, GX, CHN	OP390450
GXWZ-OP390451-2022	2022	Wuzhou, GX, CHN	OP390451
GXWZ-OP390452-2022	2022	Wuzhou, GX, CHN	OP390452
GXGL-OQ122099-2022	2022	Guilin, GX, CHN	OQ122099
