# Supplementary material for: Phylogenetic and Evolutionary Analysis of Porcine Epidemic Diarrhea Virus in Guangxi Province, China, during 2020 and 2024
Source: Viruses. 2024 Jul 14;16(7):1126. doi: 10.3390/v16071126 (PMC11281377; doi:10.3390/v16071126)
Supplement: Supplementary file 1 [file viruses-16-01126-s001.zip › Supplementary Figure S1.pdf]

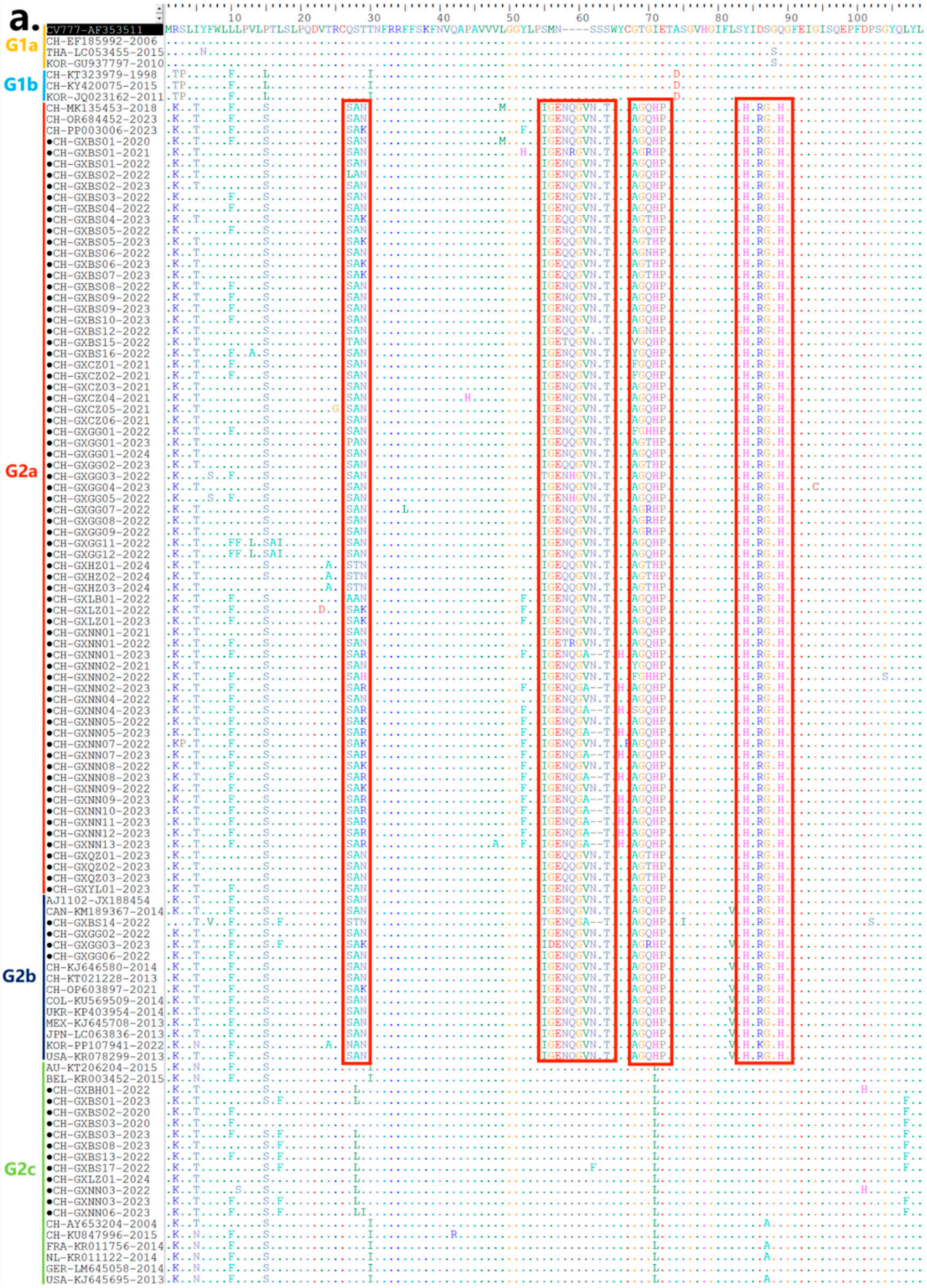
a.
CV777-AF353511
G1a
G1b
G2a
G2b
G2c

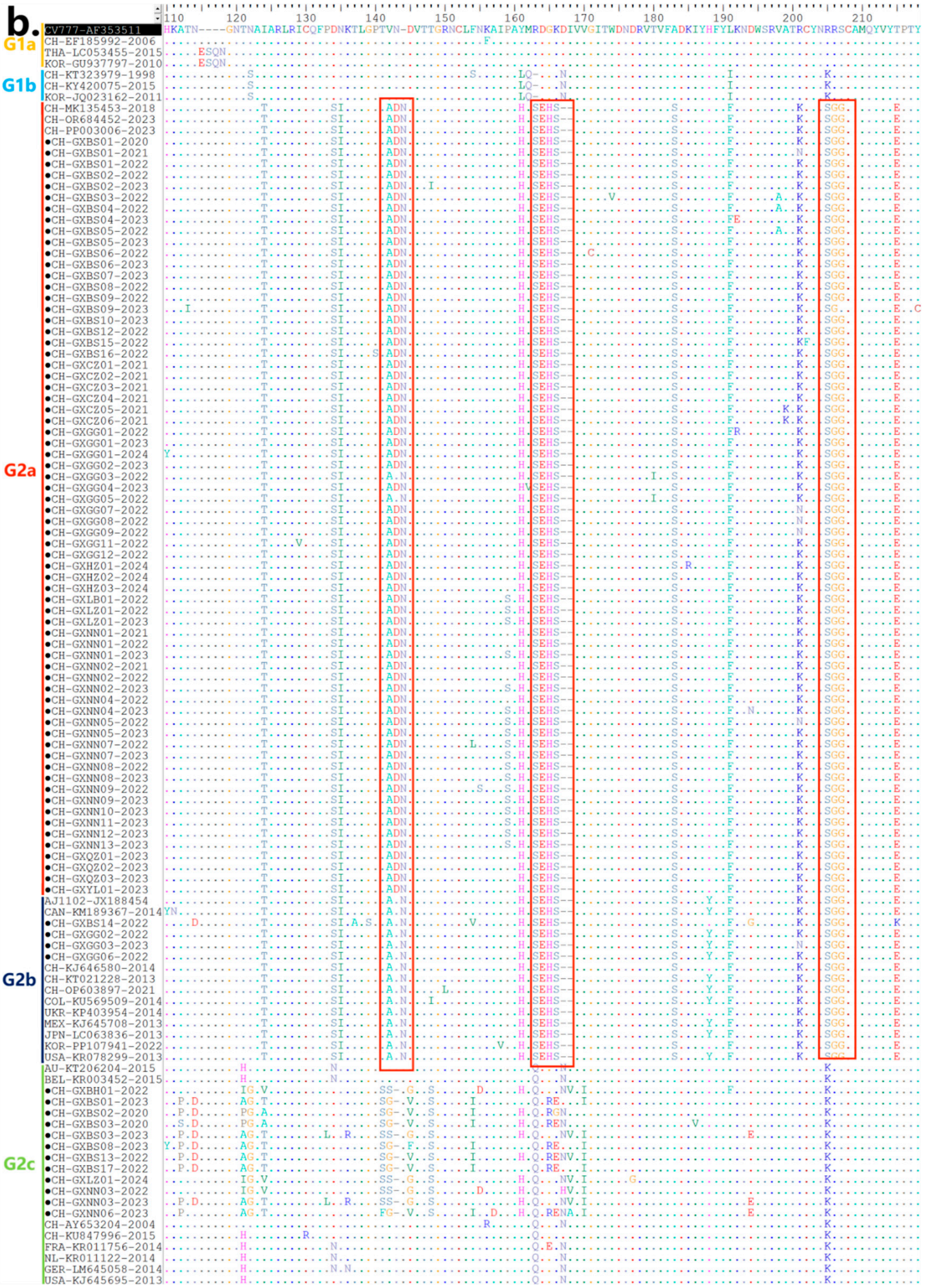
b.
G1a
G1b
G2a
G2b
G2c
CV777-AF353511

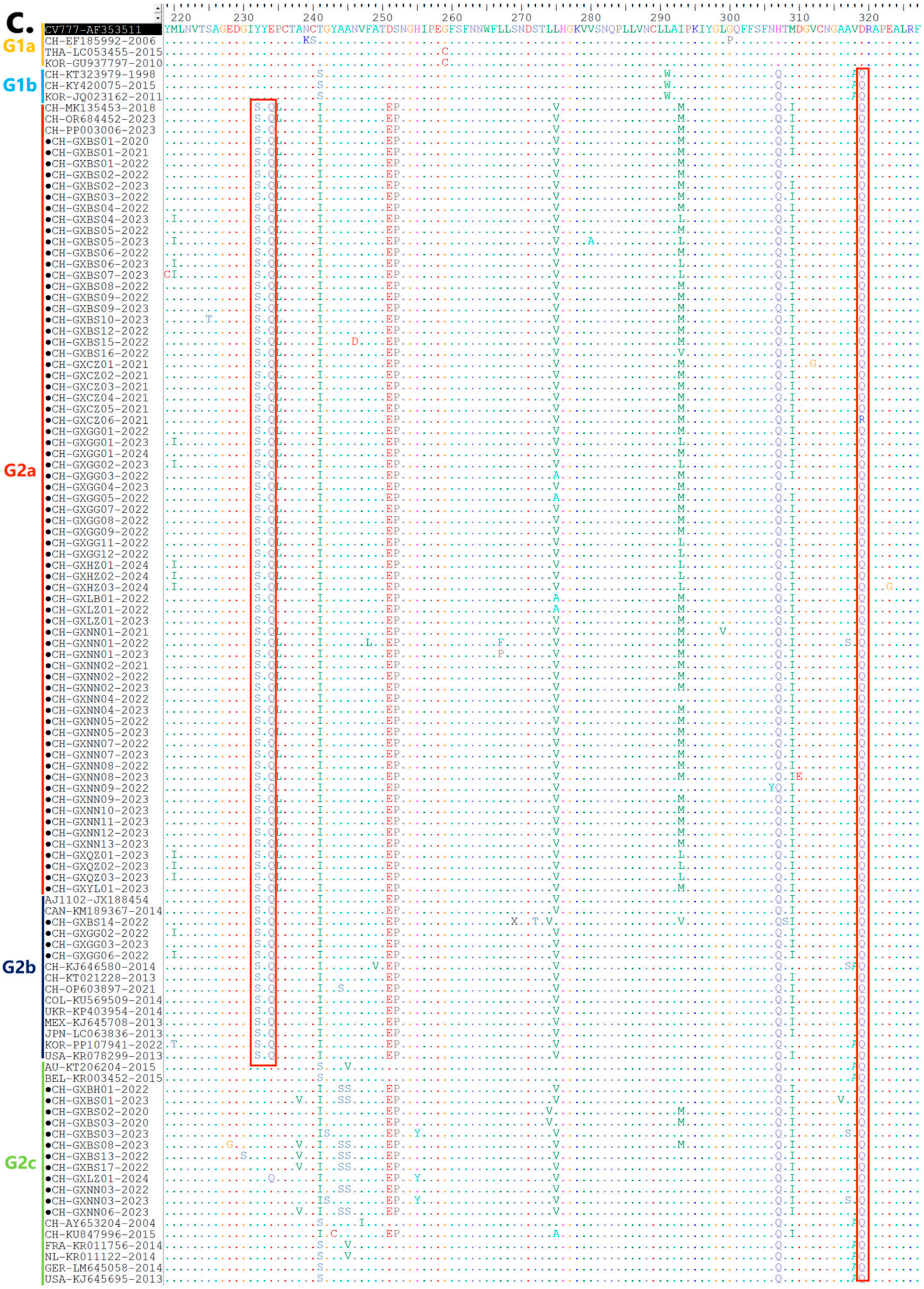

C.
220
230
240
250
260
270
280
290
300
310
320
CV777-AF353511
YMLNVTSAGEDGIYYEPCTANCTGYAANVFATDSNGHIPEGFSFNNWFLLSNDSTLLHGKVVSNQPLLVNCLLAIPKIYGLGQFFSFNHTMDGVCNGAAVDRAPEALRF
G1a
CH-EF185992-2006
THA-LC053455-2015
KOR-GU937797-2010
G1b
CH-KT323979-1998
CH-KY420075-2015
KOR-JQ023162-2011
G2a
CH-MK135453-2018
CH-OR684452-2023
CH-PP003006-2023
●CH-GXBS01-2020
●CH-GXBS01-2021
●CH-GXBS01-2022
●CH-GXBS02-2022
●CH-GXBS02-2023
●CH-GXBS03-2022
●CH-GXBS04-2022
●CH-GXBS04-2023
●CH-GXBS05-2022
●CH-GXBS05-2023
●CH-GXBS06-2022
●CH-GXBS06-2023
●CH-GXBS07-2023
●CH-GXBS08-2022
●CH-GXBS09-2022
●CH-GXBS09-2023
●CH-GXBS10-2023
●CH-GXBS12-2022
●CH-GXBS15-2022
●CH-GXBS16-2022
●CH-GXCZ01-2021
●CH-GXCZ02-2021
●CH-GXCZ03-2021
●CH-GXCZ04-2021
●CH-GXCZ05-2021
●CH-GXCZ06-2021
●CH-GXGG01-2022
●CH-GXGG01-2023
●CH-GXGG01-2024
●CH-GXGG02-2023
●CH-GXGG03-2022
●CH-GXGG04-2023
●CH-GXGG05-2022
●CH-GXGG07-2022
●CH-GXGG08-2022
●CH-GXGG09-2022
●CH-GXGG11-2022
●CH-GXGG12-2022
●CH-GXHZ01-2024
●CH-GXHZ02-2024
●CH-GXHZ03-2024
●CH-GXLB01-2022
●CH-GXLZ01-2022
●CH-GXLZ01-2023
●CH-GXNN01-2021
●CH-GXNN01-2022
●CH-GXNN01-2023
●CH-GXNN02-2021
●CH-GXNN02-2022
●CH-GXNN02-2023
●CH-GXNN04-2022
●CH-GXNN04-2023
●CH-GXNN05-2022
●CH-GXNN05-2023
●CH-GXNN07-2022
●CH-GXNN07-2023
●CH-GXNN08-2022
●CH-GXNN08-2023
●CH-GXNN09-2022
●CH-GXNN09-2023
●CH-GXNN10-2023
●CH-GXNN11-2023
●CH-GXNN12-2023
●CH-GXNN13-2023
●CH-GXQZ01-2023
●CH-GXQZ02-2023
●CH-GXQZ03-2023
●CH-GXYL01-2023
G2b
AJ1102-JX188454
CAN-KM189367-2014
●CH-GXBS14-2022
●CH-GXGG02-2022
●CH-GXGG03-2023
●CH-GXGG06-2022
CH-KJ646580-2014
CH-KT021228-2013
CH-OP603897-2021
COL-KU569509-2014
UKR-KP403954-2014
MEX-KJ645708-2013
JPN-LC063836-2013
KOR-PP107941-2022
USA-KR078299-2013
G2c
AU-KT206204-2015
BEL-KR003452-2015
●CH-GXBH01-2022
●CH-GXBS01-2023
●CH-GXBS02-2020
●CH-GXBS03-2020
●CH-GXBS03-2023
●CH-GXBS08-2023
●CH-GXBS13-2022
●CH-GXBS17-2022
●CH-GXLZ01-2024
●CH-GXNN03-2022
●CH-GXNN03-2023
●CH-GXNN06-2023
CH-AY653204-2004
CH-KU847996-2015
FRA-KR011756-2014
NL-KR011122-2014
GER-LM645058-2014
USA-KJ645695-2013

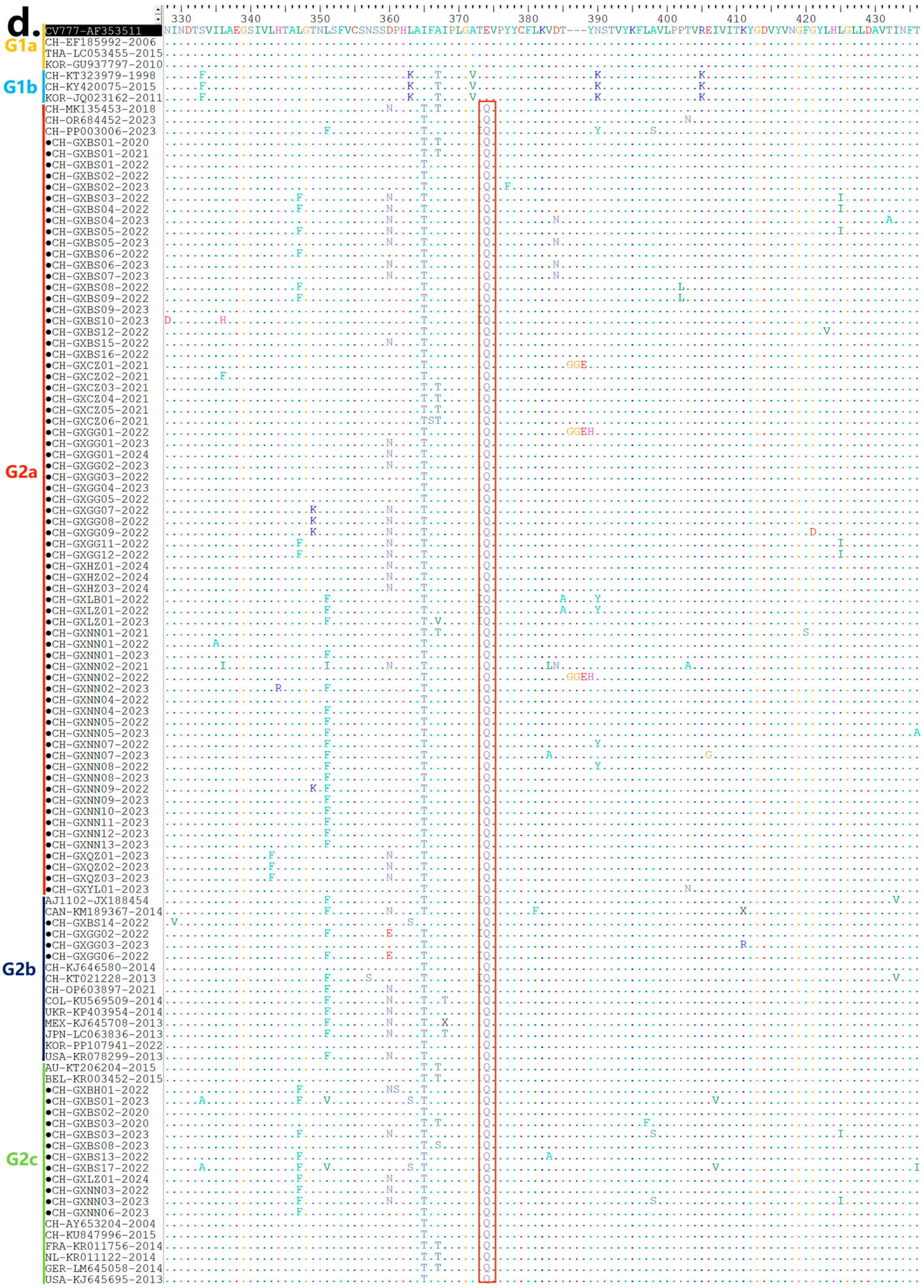
d.
330 340 350 360 370 380 390 400 410 420 430
CV777-AF353511
NINDTSVILAEGSIVLHTALGTNLSFVCSNSSDPHLAIFAIPLGATEVPYYCFLKVDT---YNSTVYKFLAVLPPTVREIVITKYGDVYVNGFGYLHLGLLDAVTINFT
G1a
G1b
G2a
G2b
G2c

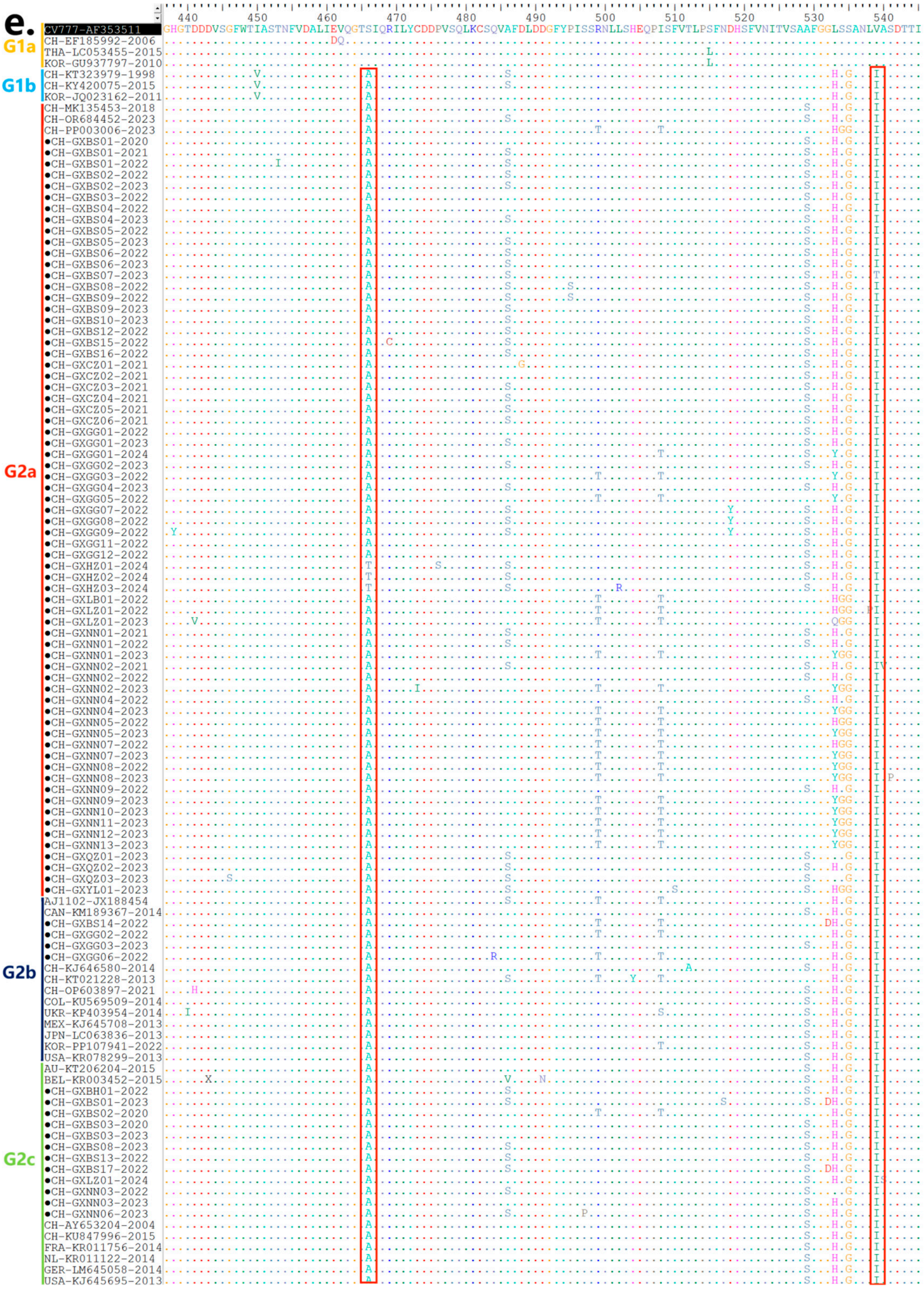
e.
G1a
G1b
G2a
G2b
G2c
CV777-AF353511
440
450
460
470
480
490
500
510
520
530
540

f.

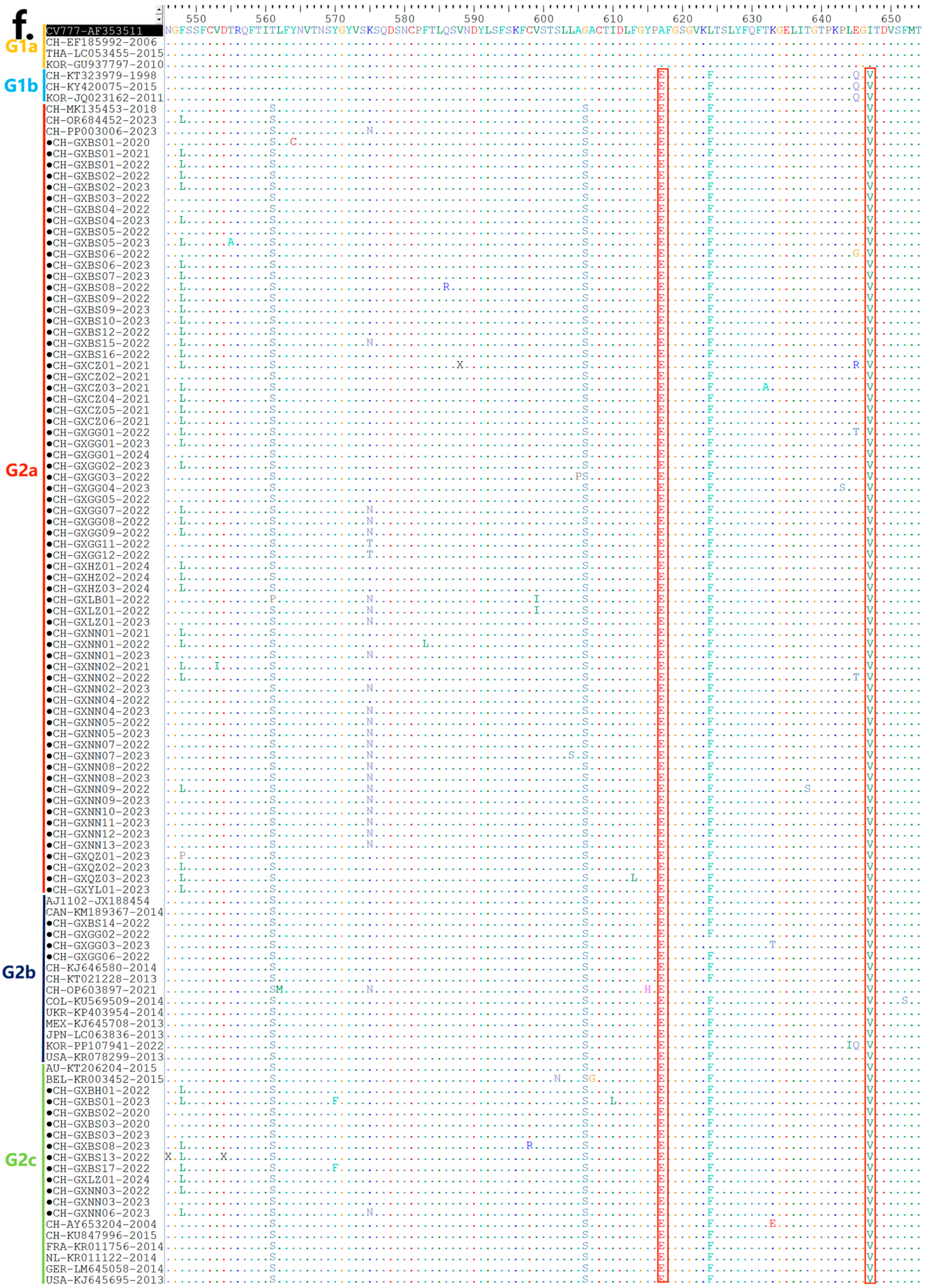

550
560
570
580
590
600
610
620
630
640
650
CV777-AF353511
G1a
G1b
G2a
G2b
G2c

g.

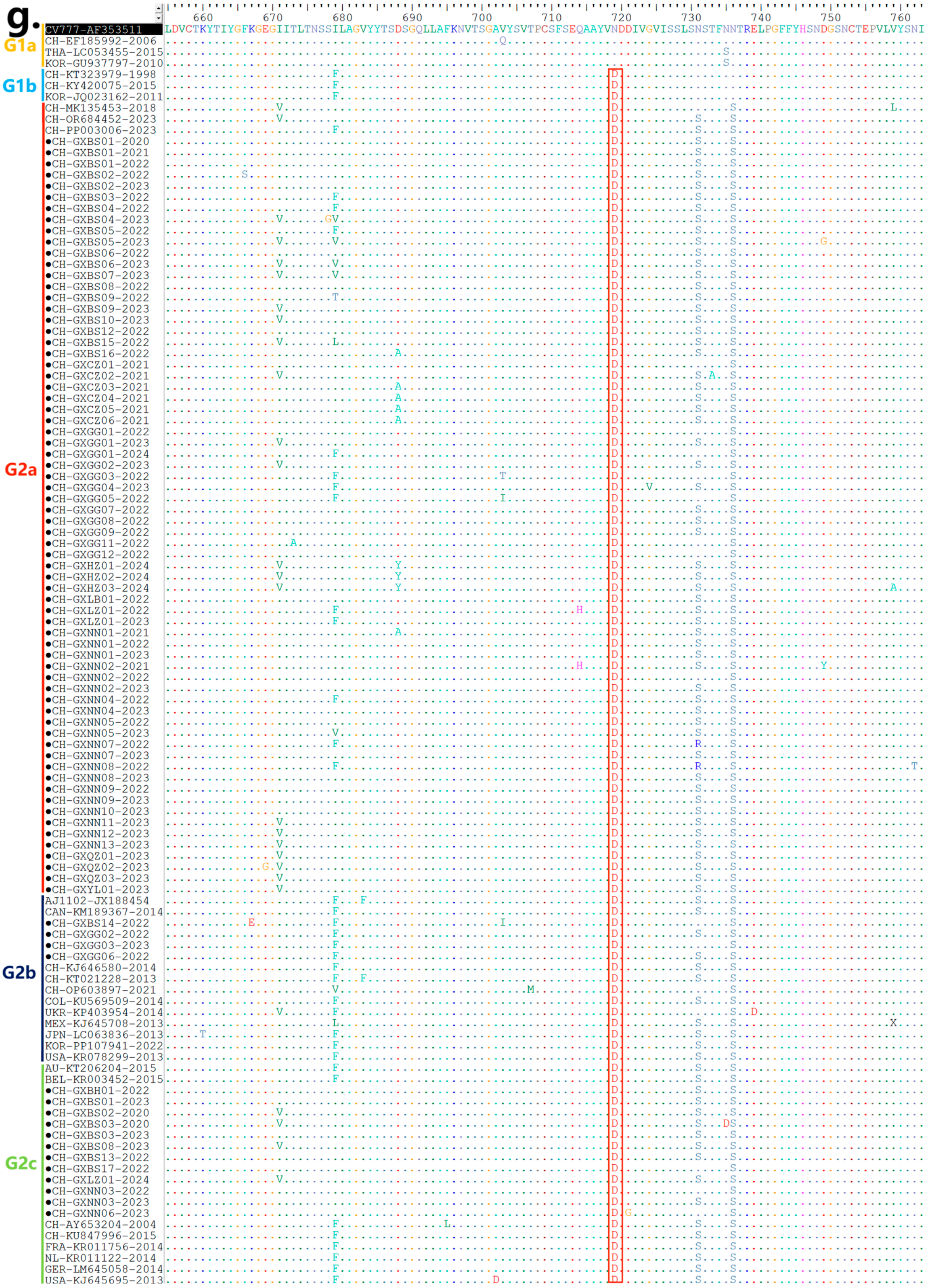

660
670
680
690
700
710
720
730
740
750
760
CV777-AF353511
LDVCTKYTIYGFKGEGIITLTNSSILAGVYYTSDSGQLLAFKNVTSGAVYSVTPCSFSEQAAYVNDDIVGVISSLSNSTFNNTRELPGFFYHSNDGSNCTEPVLVYSNI
G1a
CH-EF185992-2006
THA-LC053455-2015
KOR-GU937797-2010
G1b
CH-KT323979-1998
CH-KY420075-2015
KOR-JQ023162-2011
G2a
CH-MK135453-2018
CH-OR684452-2023
CH-PP003006-2023
•CH-GXBS01-2020
•CH-GXBS01-2021
•CH-GXBS01-2022
•CH-GXBS02-2022
•CH-GXBS02-2023
•CH-GXBS03-2022
•CH-GXBS04-2022
•CH-GXBS04-2023
•CH-GXBS05-2022
•CH-GXBS05-2023
•CH-GXBS06-2022
•CH-GXBS06-2023
•CH-GXBS07-2023
•CH-GXBS08-2022
•CH-GXBS09-2022
•CH-GXBS09-2023
•CH-GXBS10-2023
•CH-GXBS12-2022
•CH-GXBS15-2022
•CH-GXBS16-2022
•CH-GXCZ01-2021
•CH-GXCZ02-2021
•CH-GXCZ03-2021
•CH-GXCZ04-2021
•CH-GXCZ05-2021
•CH-GXCZ06-2021
•CH-GXGG01-2022
•CH-GXGG01-2023
•CH-GXGG01-2024
•CH-GXGG02-2023
•CH-GXGG03-2022
•CH-GXGG04-2023
•CH-GXGG05-2022
•CH-GXGG07-2022
•CH-GXGG08-2022
•CH-GXGG09-2022
•CH-GXGG11-2022
•CH-GXGG12-2022
•CH-GXHZ01-2024
•CH-GXHZ02-2024
•CH-GXHZ03-2024
•CH-GXLB01-2022
•CH-GXLZ01-2022
•CH-GXLZ01-2023
•CH-GXNN01-2021
•CH-GXNN01-2022
•CH-GXNN01-2023
•CH-GXNN02-2021
•CH-GXNN02-2022
•CH-GXNN02-2023
•CH-GXNN04-2022
•CH-GXNN04-2023
•CH-GXNN05-2022
•CH-GXNN05-2023
•CH-GXNN07-2022
•CH-GXNN07-2023
•CH-GXNN08-2022
•CH-GXNN08-2023
•CH-GXNN09-2022
•CH-GXNN09-2023
•CH-GXNN10-2023
•CH-GXNN11-2023
•CH-GXNN12-2023
•CH-GXNN13-2023
•CH-GXQZ01-2023
•CH-GXQZ02-2023
•CH-GXQZ03-2023
•CH-GXYL01-2023
G2b
AJ1102-JX188454
CAN-KM189367-2014
•CH-GXBS14-2022
•CH-GXGG02-2022
•CH-GXGG03-2023
•CH-GXGG06-2022
CH-KJ646580-2014
CH-KT021228-2013
CH-OP603897-2021
COL-KU569509-2014
UKR-KP403954-2014
MEX-KJ645708-2013
JPN-LC063836-2013
KOR-PP107941-2022
USA-KR078299-2013
G2c
AU-KT206204-2015
BEL-KR003452-2015
•CH-GXBH01-2022
•CH-GXBS01-2023
•CH-GXBS02-2020
•CH-GXBS03-2020
•CH-GXBS03-2023
•CH-GXBS08-2023
•CH-GXBS13-2022
•CH-GXBS17-2022
•CH-GXLZ01-2024
•CH-GXNN03-2022
•CH-GXNN03-2023
•CH-GXNN06-2023
CH-AY653204-2004
CH-KU847996-2015
FRA-KR011756-2014
NL-KR011122-2014
GER-LM645058-2014
USA-KJ645695-2013

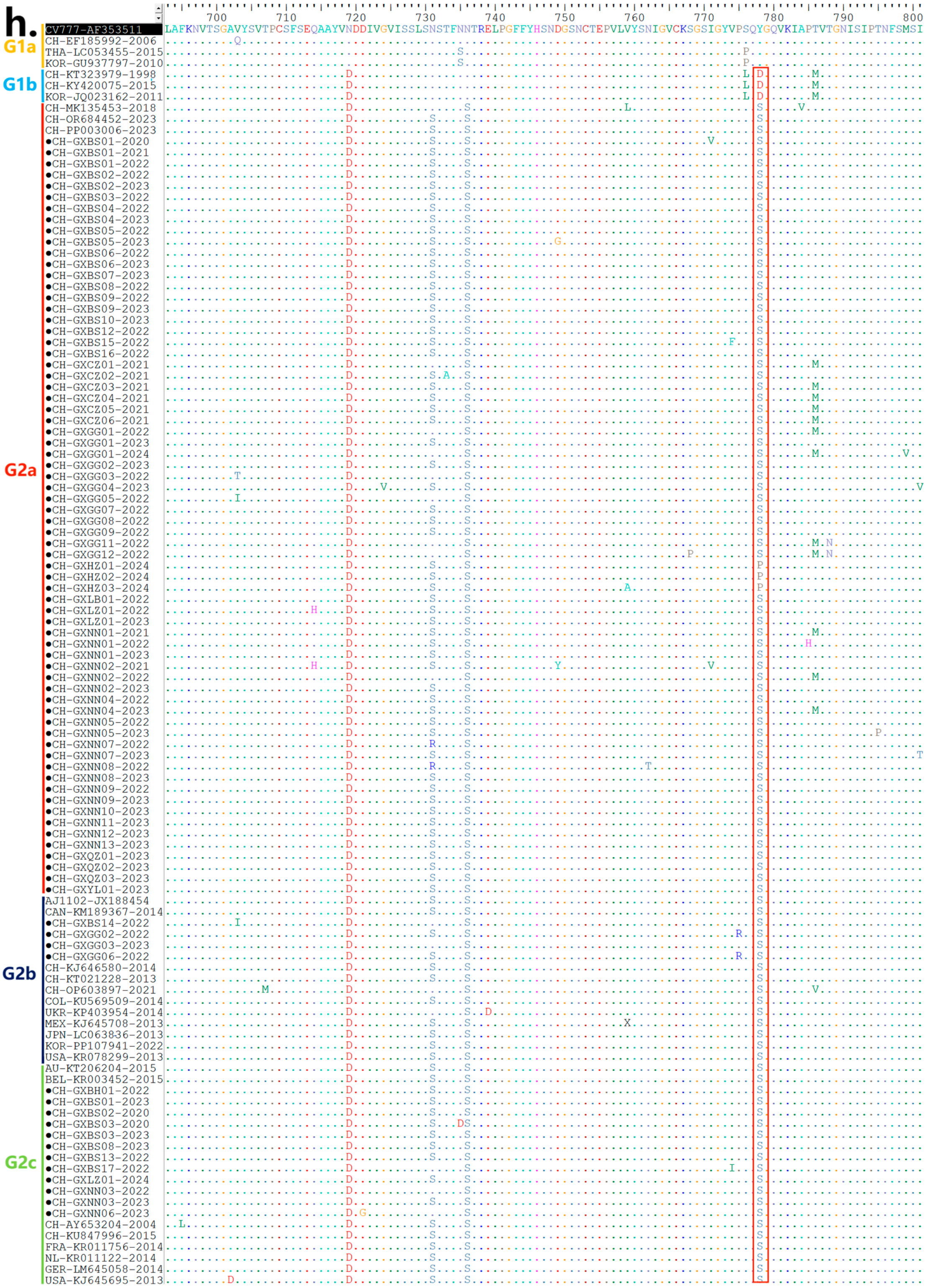

Figure S1. Amino acid difference diagrams for the PEDV S1 protein of different subgroups. The sequences were listed from (a) to (h). The sequences obtained in this study are marked with black circles.
